# The circular RNA hsa_circ_0001394 promotes hepatocellular carcinoma progression by targeting the miR-527/UBE2A axis

**DOI:** 10.1038/s41420-022-00866-0

**Published:** 2022-02-24

**Authors:** Yu Yan, Yu Nie, Chun Peng, Fuchen Xing, Saiguang Ji, Hong Liu, Chuandong Zhu

**Affiliations:** 1 Department of Oncology, The Second Hospital of Nanjing, Nanjing University of Chinese Medicine, Nanjing, 210003 China; 2 Department of Thoracic Surgery, The Second Hospital of Nanjing, Nanjing University of Chinese Medicine, Nanjing, 210003 China

**Keywords:** Tumour biomarkers, Targeted therapies, Tumour biomarkers

## Abstract

Circular RNAs (circRNAs) have been recognized as significant participants in the progression of different cancers; however, the detailed mechanisms of circRNAs in hepatocellular carcinoma (HCC) remain unclear. In this study, hsa_circ_0001394 was identified by RNA-seq analysis, and hsa_circ_0001394 was determined to be highly expressed in HCC specimens and cell lines. Patients with high expression of hsa_circ_0001394 tended to exhibit poor survival. Increased hsa_circ_0001394 expression in plasma was closely correlated with clinicopathological features including elevated vascular invasion and an advanced TNM stage, as indicated by alpha-fetoprotein (AFP) analysis. Hsa_circ_0001394 promoted the proliferation, migration, and invasion of HCC cells, whereas knockdown of hsa_circ_0001394 inhibited HCC tumorigenesis in vivo. In addition, mechanistic studies showed that miR-527 negatively interacted with hsa_circ_0001394. Furthermore, UBE2A was revealed to serve as a target of miR-527. Overall, the present study suggested that hsa_circ_0001394 may function as a sponge to promote HCC progression by regulating the miR-527/UBE2A pathway. Thus, hsa_circ_0001394 may become a promising biomarker and potential therapeutic target in HCC treatment.

## Introduction

Hepatocellular carcinoma (HCC) has emerged as a major contributor to the burden of cancer incidence globally and ranks third in cancer mortality, causing an estimated 0.83 million deaths per year [1]. Despite recent progress in HCC treatment, including immunotherapy and targeted therapy [2], the limited diagnostic methods that can be used at an early stage result in poor prognosis [3], thereby highlighting the importance of exploring HCC therapeutic targets and their detailed downstream mechanisms [4].

Circular abnormally spliced transcripts have been known to exist for 30 years [5]. Originally, the few known circular RNAs (circRNAs) were considered “junk” generated from aberrant events with little potential function [6]; however, RNA sequencing (RNA-seq) has enabled the discovery of numerous well-expressed circRNAs, which has attracted widespread interest [7]. Formed by back splicing of an upstream 5′ site and a downstream 3′ site of the same transcript, circRNAs form a cluster of noncoding RNAs with covalent closed-loop structures [8]. CircRNAs consist of mainly exons [9] or introns [10], and they generally exist in the cytoplasm, in contrast to their corresponding linear RNAs, due to their lack of polyadenylated (poly(A)) tails and 5′ caps [11]. Due to their ring loop structures, circRNAs are more stable than their linear counterparts [12]. CircRNAs have been reported to participate in various diseases [13], including diabetes mellitus [14], nonalcoholic steatohepatitis (NASH) [15], cardiovascular diseases [16], and cancers [17]. Recent findings have widely highlighted the contributions of circRNAs to tumorigenesis such as in breast cancer [18], lung cancer [19], gastric cancer [20], and colon cancer [21]. Numerous circRNAs have been found to be associated with HCC, including circCSMARCA5 [22], circRHDT1 [23], circASAP1 [24] and circMTO1 [25]. CircRNAs exert their functions by acting as competing endogenous RNAs (ceRNAs) of microRNAs (miRNAs) [26], interacting with RNA-binding proteins (RBPs), and modifying parental gene transcription [27]. MiRNAs are small noncoding RNAs of ~22 nucleotides in length [28]. The dysregulation of miRNAs contributes to human diseases, particularly cancers, by disrupting the regulation of downstream mRNAs [29]. Targeting miRNAs, which can serve as either tumor inhibitors or oncogenes by interacting with circRNAs, have demonstrated promise in preclinical studies [30]. For instance, circRNA-5692 is downregulated in HCC tissues and can suppress the malignant behaviors of HCC by targeting oncogenic miR-328-5p to regulate DAB2IP [31]. Another study indicated that circRNA-104718 enhances HCC progression by sponging the miR-218-5p tumor inhibitor by targeting TXNDC5 [32]. Research on the interactions between circRNAs and miRNAs in cancer has identified circRNAs as potential biomarkers for therapy. However, the complex downstream mechanisms remain unclear.

In our study, we selected the novel circRNA hsa_circ_0001394 through RNA-seq of three pairs of HCC tissues and adjacent normal tissues. Hsa_circ_0001394 was found to be significantly upregulated in HCC cell lines, tissues, and plasma compared to normal samples, and its levels were correlated with HCC prognosis. Overall, the present study aimed to identify the biological function of hsa_circ_0001394 in HCC progression and to further explore its potential targets and molecular mechanisms. The results may provide insights that will aid the development of a promising diagnostic biomarker and therapeutic target for HCC.

## Materials and methods

### HCC tissue specimens

Fifty pairs of HCC tissues and adjacent normal tissues were obtained from patients during surgical resection without chemotherapy or radiotherapy (from 2017 to 2021) at the Second Hospital of Nanjing (Nanjing, China). Every patient provided prior written informed consent, and this study was approved by the Ethics Committee of the Second Hospital of Nanjing (2017-LY-kt038) and performed according to the Declaration of Helsinki.

### Cell culture

Human HCC cell lines (Hep3B, HCCLM3, SMMC7721, MHCC97H, HepG2, and Huh7), a normal liver cell line (LO2), and 293 T cells were obtained from the Cell Bank of the Chinese Academy of Sciences (Shanghai, China). Cells were cultured in high-glucose Dulbecco’s modified Eagle’s medium (DMEM) (Gibco, Carlsbad, CA, USA) containing 10% fetal bovine serum (FBS) and 1% penicillin-streptomycin (Gibco, Carlsbad, CA, USA) at 37 °C under 5% CO_2_.

### Transfection and stable cell line construction

Short hairpin RNA targeting hsa_circ_0001394 (sh-circRNA), short hairpin RNA-related negative controls (sh-NC), pcDNA3.1(+) circRNA vector for hsa_circ_0001394 overexpression, overexpression-related negative controls (Lv-NC), miR-527 mimics, miR-527 inhibitors (miR-527 inh), and related control plasmids, including NC mimics (miR-NC) and NC inhibitors (inh-NC) were synthesized by RiboBio (Guangzhou, China). The vectors were transfected with Lipofectamine 3000 (Invitrogen, Carlsbad, CA, USA).

### Plasmid construction

pCMV-Myc plasmids expressing UBE2A were constructed by cloning UBE2A PCR products, which were amplified from HeLa cell cDNA into pCMV-Myc vectors. Small interfering RNAs (siRNAs) against UBE2A were designed by the GenePharm company. Transfection of siRNA into HeLa cells was performed according to the manufacturer’s protocol (Invitrogen). Briefly, 3 μg of siRNAs were transfected with 8 μl Lipofectamine 3000 (Invitrogen) per well of a 6-well plate.

### RNA isolation and real-time quantitative PCR (qRT-PCR)

Total RNA was isolated from cell lines and tissues with TRIzol (Invitrogen, Carlsbad, CA, USA) following the manufacturer’s instructions. The extracted RNA was reverse-transcripted into complementary DNA using a PrimeScript RT Kit (Takara, Dalian, China). Alternatively, a RiboBio reverse transcription kit (Guangzhou, China) was used to reverse-transcribe miRNAs under the recommended conditions. qRT-PCR was run on an ABI7500 instrument using SYBR Green Master Mix (Takara, Shanghai, China). GAPDH and U6 were selected to normalize the expression levels of mRNAs and miRNAs, respectively. The relative expression was calculated with the 2^−△△CT^ algorithm. All primers used in this experiment are shown in Table 1.

**Table 1:** Primers used in the present study.

Gene	Primer sequence
miR-527	forward:5′-CTCAAGCTGTGACTGCAAAGG-3′
	reverse:5′-AATTCACCAAAGGGAAGCACT-3′
U6	forward:5′-CTCGCTTCGGCAGCACA-3′
	reverse: 5′-AACGCTTCACGAATTTGCG-3′
hsa_circ_0001394	forward:5′-TGTGTTCATGAAGCTGAGGAGG-3′
	reverse:5′-CGACCATCCAGAATACCCATGA-3′
UBE2A	forward:5′-GGAGTCCAACCTATGATGTGTCT-3′
	reverse:5′-CATATTCCCGTTTGTTCTCCTGG-3′
GAPDH	forward:5′-CCGGGAAACTGTGGCGTGATGG-3′
	reverse: 5′-AGGTGGAGGAGTGGGTGTCGCTGTT-3′

### RNase R treatment

Hsa_circ_0001394 extracted from the HCC cell lines was incubated for 30 min at 37 °C with RNase R (4U/mg). The relative expression levels of hsa_circ_0001394 and related linear RNA were assessed by qRT-PCR.

### Nuclear–cytoplasmic fractionation and localization

The cytoplasmic RNAs and nuclear RNAs of the HCC cell lines were isolated with a PARIS kit (Life Technologies) following the manufacturer’s instructions. qRT-PCR was conducted to detect the localization of hsa_circ_0001394.

### Cell Counting Kit-8 (CCK-8) assay

Transfected HCC cells were seeded into 96-well plates at a density of 2 × 10^3^ cells/well. CCK-8 reagent (Dojindo, Japan) was added to each well, and HCC cell viability was assessed at three time points (24, 48, and 72 h). After incubation with 10 μl of CCK-8 reagent for 2 h, the absorbance of each well at 450 nM was measured using a Varioskan LUX (Thermo Fisher, CA, USA).

### Colony formation assay

Transfected HCC cells were plated into six-well plates and incubated under 5% CO_2_ at 37 °C. After 2 weeks, the cells were washed with phosphate-buffered saline (PBS) and fixed with 4% paraformaldehyde for 20 min. Crystal violet was used to stain the cells. Each colony containing at least 50 cells under a microscope was counted as valid.

### 5-Ethynyl-2′-deoxyuridine (EdU) assay

An EdU assay kit (RiboBio, China) was utilized to evaluate cell proliferation. Transfected HCC cells were plated into 24-well plates and incubated for 24 h. After adhesion, 60 μM EdU reagent was added, and cells were then cultured for 3 h. Cells were then stained with Apollo Dye Solution. The percentage of EdU-positive cells was quantified using fluorescence microscopy (Nikon, Shanghai).

### Wound-healing assay

HCC cells (1 × 10^6^ cells/well) were seeded into 6-well plates and grown to 90% confluence under 5% CO_2_ at 37 °C. Cells were wounded by scratching down the middle of the plate with a 200 μl pipette tip. After three washes with PBS, serum-free medium was added to culture the cells. Images were acquired at 0 and 24 h after injury at the same site.

### Transwell assay

After suspension with 200 μl of serum-free medium, 2 × 10^4^ HCC cells were placed into the upper compartment of a Transwell chamber (Corning, USA), and 600 μl of DMEM with 10% serum was added into the lower compartment as a chemoattractant. After 24 h, the invaded cells were treated with 4% paraformaldehyde and crystal violet for 20 min for fixation and staining, respectively. Images were acquired using a Nikon microscope.

### Luciferase reporter assay

Two hundred ninety-three T cells were plated onto 24-well plates. After treatment with miR-527 mimics or negative controls, 293 T cells were transfected with vectors containing wild-type (WT) or mutant-type (MUT) hsa_circ_0001394 and UBE2A (Promega, WI, USA) using Lipofectamine 3000 (Invitrogen, Carlsbad, CA, USA). After 48 h, the luciferase activity of each well was detected with a luciferase reporter assay system (Promega).

### RNA pull-down assay

The biotinylated hsa_circ_0001394 probe synthesized by RiboBio (Guangzhou, China) was used to pull-down miRNAs. Hep3B and Huh7 cells were crosslinked with glutaraldehyde, equilibrated in glycine buffer, and treated with lysis buffer prior to scraping from culture plates. The cell samples were then sonicated to shear DNA and centrifuged. The supernatants were transferred into 1 ml tubes, and 10 μl of each sample was separately saved for input analysis. The hsa_circ_0001394 probe was incubated with C-1 magnetic beads (Life Technologies) at 25 °C for 2 h to generate probe-coated beads. Subsequently, the lysates were incubated with the hsa_circ_0001394 probe or an oligo probe at 4 °C overnight, forming specific probe-miRNA complexes. The RNA complexes that bound to the beads were eluted with washing buffer, incubated to reverse the glutaraldehyde cross-linking, and then extracted with TRIzol. The relative expression levels of the pulled-down miR-527 were quantified using qRT-PCR. The hsa_circ_0001394 probe sequence is TCCAAGGAGAAACTTGCAAA (5′-3′). The control probe sequence used in this assay is CACGTAGTAACGAGACAATA (5′-3′).

### Western blotting

HCC tissues and cells were lysed with RIPA buffer containing phenylmethanesulfonyl fluoride on ice for 20 min and quantified using a BCA protein assay kit. The proteins were separated via 10% sodium dodecyl sulfate–polyacrylamide gel electrophoresis (SDS-PAGE) and transferred onto polyvinylidene difluoride (PVDF) membranes (Millipore, Bedford, MA, USA) and the membranes were blocked with 5% nonfat milk powder for 2 h. The membranes were then incubated with primary antibodies at 4 °C overnight. Afterward, the secondary antibody was added, and the membranes were incubated for 2 h at room temperature. Subsequently, an enhanced chemiluminescence (ECL) system (Thermo Fisher Scientific) was used to evaluate the protein expression levels. The primary antibodies used in this experiment included anti-UBE2A (Abcam, #ab31917, 1:1000), anti-E-cadherin (Abcam, #ab1416, 1:1000), anti-N-cadherin (Abcam, #ab98952, 1:1000), anti-vimentin (Abcam, #ab92547, 1:1000), anti-snail (Abcam, #ab216347, 1:1000), anti-MDM2 (Abcam, #ab16895, 1:1000), anti-p53 (Abcam, #ab32389, 1:2000), anti-Bcl-2 (Abcam, #ab185002, 1:1000), anti-Bax (Abcam, #ab32503, 1:1000) and anti-GAPDH (Abcam, #ab181602, 1:1000).

### Immunohistochemistry (IHC)

After fixation in formalin, the tissues were dehydrated and sliced into 4-μm paraffin sections. Hematoxylin–eosin (HE) staining was conducted with standard procedures. The sections were incubated with anti-Ki67 (1:1000, Abcam, Cambridge, UK) at 4 °C overnight. After the sections were washed with PBS, HRP-conjugated polyclonal secondary antibodies were added followed by incubation at room temperature for 30 min.

### Tumor xenografts

Five-week-old BALB/C nude mice were divided into two groups at random (five mice per group). Huh7 cells transfected with sh-circRNA or a mock vector were injected subcutaneously into the mice. All mice were sacrificed 1 month later. The tumor size was measured at 5-day intervals, and the tumor was weighed at the end of the experiment. The experimental animals were maintained in accordance with the guidelines of the National Institutes of Health (NIH) and with the approval of the Ethics Committee of the Second Hospital of Nanjing.

### Statistical analysis

All values are presented as the mean±standard deviation (mean ± SD) from at least three independent experiments. All data were analyzed using SPSS 22.0 (SPSS, Chicago, IL, USA) and GraphPad Prism 9.0 (GraphPad Software, Inc., CA, USA). The variance is similar between the groups that are being statistically compared. Student’s *t*-test or the *χ*^2^-test was conducted to compare various groups, and Fisher’s exact test was performed to evaluate the relationships between hsa_circ_0001394 expression and clinical features. The overall survival and statistical correlations were determined using Kaplan–Meier analysis and Pearson’s correlation analysis, respectively. *P*-values < 0.05 were considered statistically significant.

## Results

### Hsa_circ_0001394 is upregulated in HCC specimens and cell lines

**Fig. 1:**
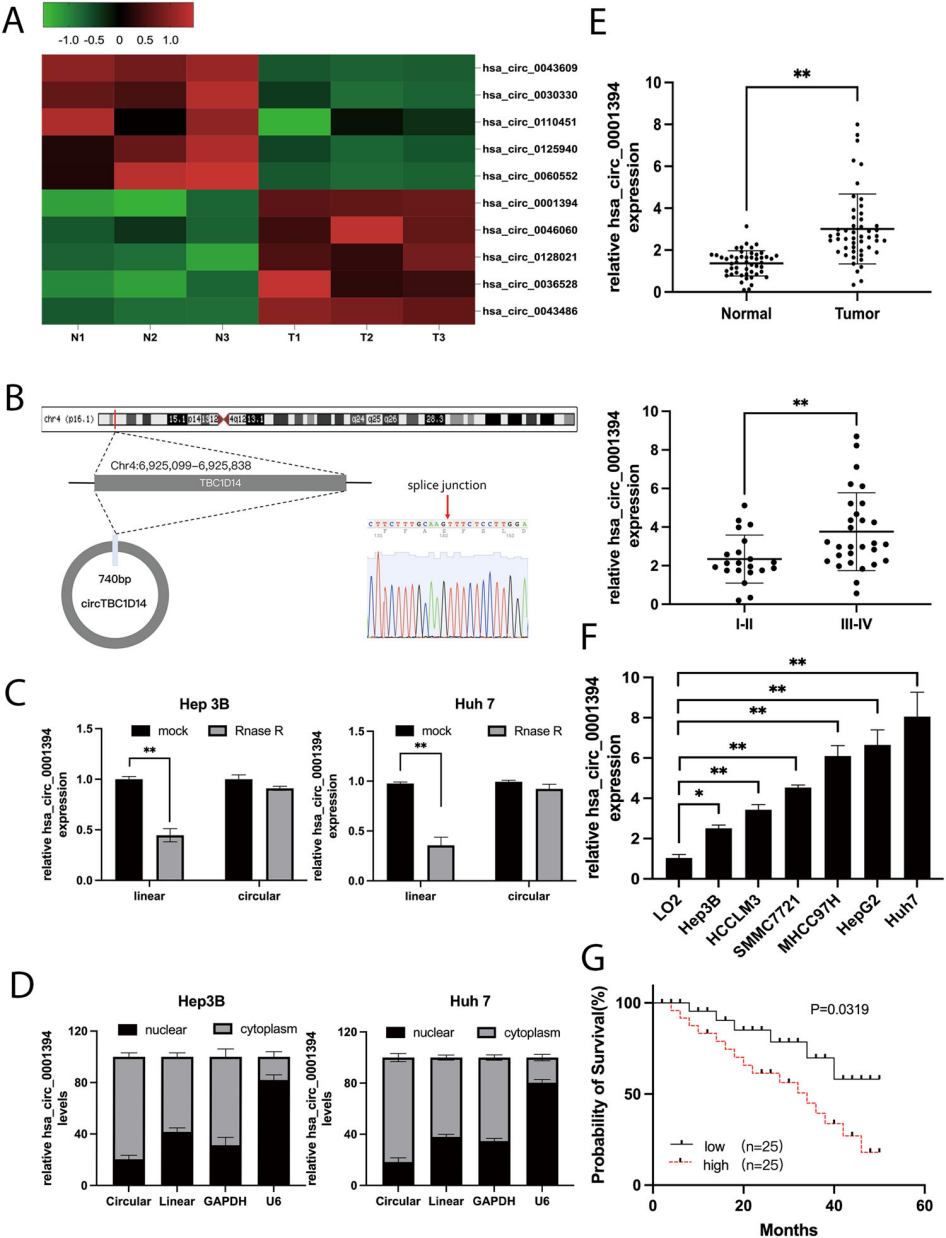
Upregulation of hsa_circ_0001394 in HCC specimens and cell lines. **A** Heatmap illustrating the top 5 high and low expressed circRNAs between three pairs of HCC specimens (right) and matched healthy controls (left). **B** The spliced mature length of hsa_circ_0001394 corresponding to the TBC1D14 gene is 740 bp and the head-to-tail splicing of hsa_circ_0001394 was confirmed by Sanger sequencing. **C** The expression of hsa_circ_0001394 and TBC1D14 mRNA in both Hep3B and Huh7 cells was evaluated by qRT-PCR treated with or without RNase R. Hsa_circ_0001394 rather than linear TBC1D14, resisted digestion by RNase R. **D** Assessment of the predominant localization of hsa_circ_0001394 using qRT-PCR. Hsa_circ_0001394 was mainly located in the cytoplasm. **E** Evaluation of relatively various expression levels of hsa_circ_0001394 between 50 pairs of HCC specimens and matched paracarcinoma tissues according to qRT-PCR. The expression of hsa_circ_0001394 was higher in HCC specimens than in control specimens. Expression of hsa_circ_0001394 in different clinical stages of HCC samples according to qRT-PCR. The expression of hsa_circ_0001394 in early stage (I–II) HCC was markedly lower than that in advanced-stage (III–IV) HCC. **F** Assessment of relative hsa_circ_0001394 expression in six HCC cell lines (Hep3B, HCCLM3, SMMC7721, MHCC97H, HepG2, and Huh7) compared to that in normal liver cells (LO2) according to qRT-PCR. **G** The 50 HCC samples were divided into high and low groups according to hsa_circ_0001394 expression. Kaplan–Meier survival analysis indicated that the group with high hsa_circ_0001394 expression had a lower overall survival (*p* = 0.0319) than the group with low hsa_circ_0001394 expression. All values are presented as mean ± SD from three independent experiments with similar results. (*p < 0.05, **p < 0.01, ***p < 0.001).

RNA-seq analysis of three pairs of HCC and matched adjacent normal tissues was conducted to determine the expression profiles of dysregulated circRNAs. The top five significantly upregulated and top five significantly downregulated circRNAs with at least a 2-fold change and a *P*-value < 0.05 are displayed in a heatmap (Fig. 1A). Hsa_circ_0001394, an upregulated circRNA, was selected for subsequent analysis, and this circRNA has not been reported previously. Derived from TBC1D14 parental gene, spliced hsa_circ_0001394 (termed circTBC1D14) is 740 base pairs in length and located at Chr4:6 925099–6925838. The head-to-tail back-splicing structure was confirmed by Sanger sequencing (Fig. 1B). To examine the stability of hsa_circ_0001394, we treated HCC cell lines with RNase R. The results showed that hsa_circ_0001394 remained more stable than the linear transcript due to its loop structure, while linear TBC1D14 degraded significantly due to a lack of resistance to RNase R (Fig. 1C). Nuclear–cytoplasmic fractionation was performed to confirm the localization of hsa_circ_0001394 using qRT-PCR. Hsa_circ_0001394 was expressed mainly in the cytoplasm instead of in the nucleus in HCC cells (Fig. 1D). GAPDH and U6 were used as controls. The expression of hsa_circ_0001394 was higher in HCC specimens than in control specimens, as determined by qRT-PCR. The expression of hsa_circ_0001394 in early stage (I–II) HCC was markedly lower than that in advanced-stage (III–IV) HCC (Fig. 1E, *n* = 50). Hsa_circ_0001394 was expressed at higher levels in six HCC cell lines (Hep3B, HCCLM3, SMMC7721, MHCC97H, HepG2, and Huh7) compared to in normal liver cells (LO2) (Fig. 1F). Compared to other HCC cells, Hep3B and Huh7 cells presented the lowest and highest hsa_circ_0001394 levels, respectively. Therefore, Hep3B and Huh7 cells were selected for further study. Additionally, we explored the relationship between hsa_circ_0001394 expression and HCC prognosis. Patients with lower hsa_circ_0001394 expression tended to have a higher possibility of survival (Fig. 1G). To further investigate the correlation between hsa_circ_0001394 expression and various clinical characteristics, we collected 62 HCC plasma samples and divided them into high-hsa_circ_0001394-expression and low-hsa_circ_0001394-expression groups according to the qRT-PCR results. The data revealed that hsa_circ_0001394 expression in preoperative blood was correlated with the AFP level (*p* = 0.0117), tumor differentiation (*p* = 0.0039), and vascular invasion (*p* = 0.0137), while there was no significant difference in age, sex, tumor size, liver cirrhosis, or hepatitis virus between the expression groups (*p* > 0.05) (Table 2). Overall, we found that hsa_circ_0001394 was highly expressed in HCC cell lines, specimens, and plasma samples, particularly in the cytoplasm, and that high hsa_circ_0001394 expression was related to poor HCC survival.

**Table 2:** Correlation between circTBC1D14 expression and clinicopathological features in HCC plasma samples (*n* = 62).

Clinical features	circTBC1D14 expression		*P*-value
	High (≥median)	Low (<median)	
Case	49	13	
*Age(year)*			0.6111
<60	16	13	
≥60	15	18	
*Gender*			0.7962
Male	20	17	
Female	12	13	
*Tumor size*			0.3087
<5 cm	14	17	
≥5 cm	19	12	
*AFP(ng/ml)*			0.0117
<400	12	13	
≥400	30	7	
*Tumor differentiation*			0.0039
I–II	10	12	
III–IV	33	7	
*Vascular invasion*			0.0137
Yes	41	3	
No	12	6	
*Liver cirrhosis*			0.5049
Yes	36	10	
No	11	5	
*Hepatitis virus*			0.7552
Positive	28	6	
Negative	22	6

### Hsa_circ_0001394 promotes the proliferation, migration, and invasion of HCC cells in vitro

**Fig. 2:**
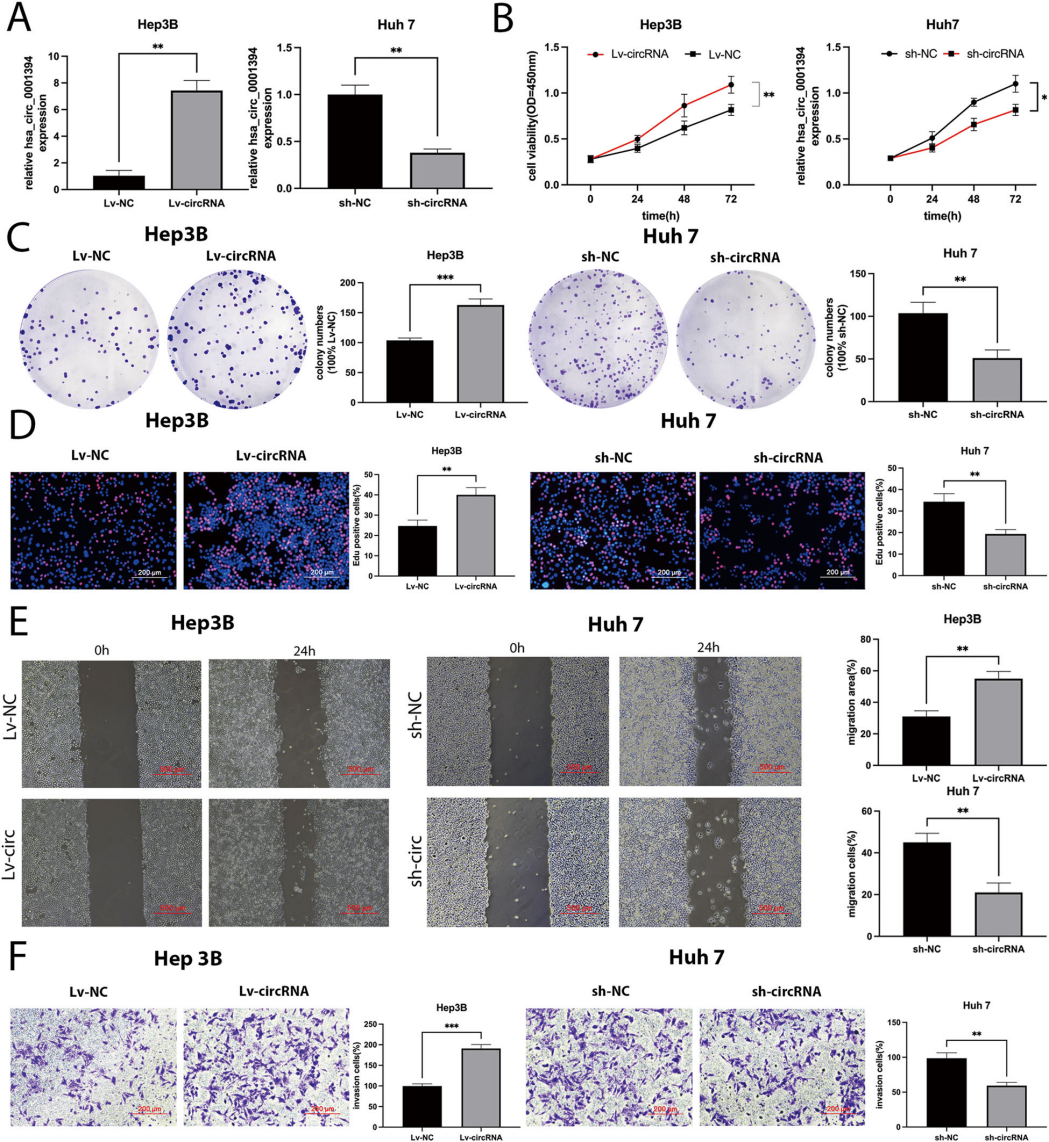
Hsa_circ_0001394 promotes the proliferation, migration, and invasion of HCC cells in vitro. **A** Relative expression of hsa_circ_0001394 overexpression and knockdown in Hep3B and Huh7 cells was detected by qRT-PCR. The overexpression system in Hep3B cells was constructed after stable transfection with pcDNA3.1(+) circRNA vectors for overexpressing hsa_circ_0001394 (Lv-circ). Lv-NC served as control plasmids of Lv-cric. The knockdown system in Huh7 cells was constructed after stable transfection with the short hairpin RNA target hsa_circ_0001394 (sh-circ). Sh-NC served as control plasmids of sh-circ. **B** The growth of Hep3B cells transfected with Lv-hsa_circ_0001394 (Lv-circ) and Huh7 cells transfected with sh-hsa_circ_0001394 (sh-circ) was evaluated using a CCK-8 assay. **C** Colony formation experiments showed that hsa_circ_0001394 promoted the colony formation activity of HCC cells. **D** Comparison of HCC cells transfected with Lv-hsa_circ_0001394 (Lv-circ) or sh-hsa_circ_0001394 (sh-circ) and corresponding control groups in EdU assays. **E** Wound-healing assays were performed to detect the effect of hsa_circ_0001394 on HCC cell migration ability. **F** Transwell assays were used to measure the effect of hsa_circ_0001394 on HCC invasion capacity. Lv-circ, pcDNA3.1(+) circRNA vector for hsa_circ_0001394 overexpression; Lv-NC, negative controls of Lv-circ; sh-circ, short hairpin RNA targeting hsa_circ_0001394; sh-NC, negative controls of sh-circ. All values are presented as mean ± SD from three independent experiments with similar results. (*p < 0.05, **p < 0.01, ***p < 0.001).

To explore the detailed function of hsa_circ_0001394 in HCC, we constructed hsa_circ_0001394-overexpressing Hep3B cells by transfecting Hep3B cells with Lv-circRNA, and Lv-NC was used as a control. In addition, an hsa_circ_0001394 knockdown system was established by transfecting sh-circRNA into Huh7 cells and sh-NC was used as a control. The efficiency of transfection in Hep3B and Huh7 cells was examined using qRT-PCR (Fig. 2A). Then, CCK-8 assays were then conducted to determine cell viability by measuring the absorbance at a wavelength of 450 nm. Transfection of HCC cells with Lv-circRNA promoted cell viability, while knockdown of hsa_circ_0001394 had the opposite effect (Fig. 2B). Next, colony formation experiments were used to detect the proliferation ability of HCC cells. Colony formation ability was markedly increased by overexpression of hsa_circ_0001394; however, silencing of hsa_circ_0001394 reduced colony formation ability (Fig. 2C). Subsequently, EdU assays were performed, which revealed that Lv-circRNA-transfected HCC cells had a greater percentage of EdU-positive cells than Lv-NC-transfected cells. In contrast, the percentage of EdU-positive cells in the hsa_circ_0001394-knockdown group was lower than that in the control group (Fig. 2D). Wound-healing assays demonstrated that hsa_circ_0001394- overexpressing HCC cells had greater mobility than control cells, while the knockdown cells were less mobile than control cells (Fig. 2E). The effect of hsa_circ_0001394 on invasion ability was determined by Transwell assay. Transfection of HCC cells with Lv-circRNA significantly promoted the proliferation, migration and invasion of the HCC cells (Fig. 2F).

### Hsa_circ_0001394 sponges miR-527

**Fig. 3:**
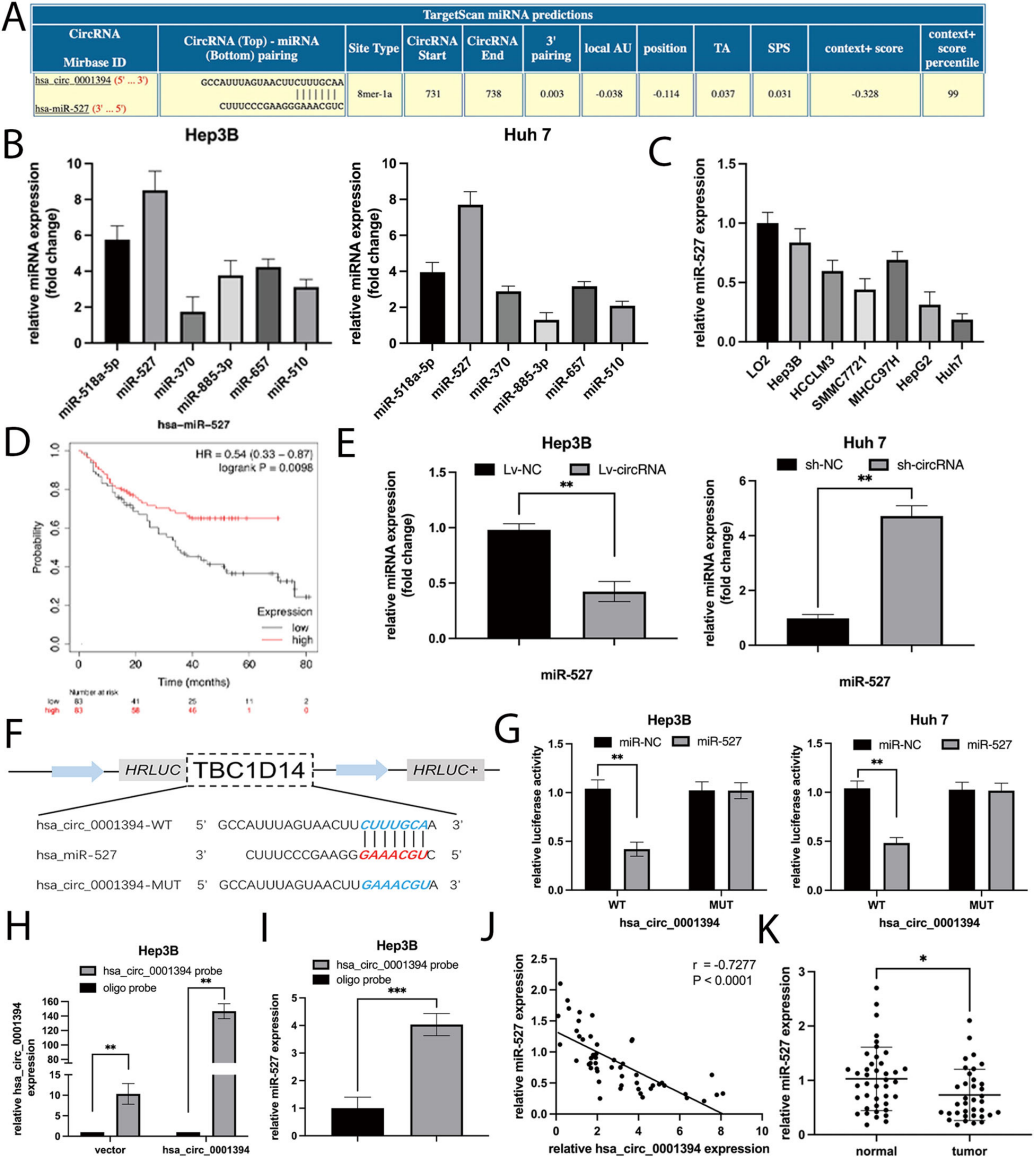
Hsa_circ_0001394 serves as a sponge of miR-527. **A** Circular RNA Interactome predicted potential binding sites for the 3′UTR of hsa_circ_0001394 and miR-527. **B** CircRNA pull-down experiments were performed in Hep3B and Huh7 cells using a specific biotin-labeled hsa_circ_0001394 probe. Multiple miRNAs were pulled down by hsa_circ_0001394. Relative abundances of six miRNA candidates were detected by qRT-PCR. **C** The expression of miR-527 in six HCC cells and normal liver cells was examined by qRT-PCR. MiR-527 was downregulated in HCC cells. Hep3B and Huh7 cells exhibited the highest and lowest miR-527 expression respectively, among the six HCC cell lines. **D** Analysis of the relationship between the survival probability and miR-527 levels in patients using a database. HCC patients with higher miR-527 expression tended to have longer survival. **E** Relative expression levels of miR-527 in HCC cell lines transfected with Lv-NC or Lv-circ and sh-NC or sh-circ according to qRT-PCR. **F** Schematic illustration of potential binding sites of miR-527 with WT (wild-type) or MUT (mutated-type) hsa_circ_0001394. **G** Relative luciferase activities were evaluated to confirm the binding sites after cotransfection of hsa_circ_0001394-MUT, hsa_circ_0001394-WT, miR-NC, and miR-527 mimics into Hep3B and Huh7 cells. **H** Verification of the significantly upregulated pull-down efficiency of hsa_circ_0001394 probe expression in lysates of Hep3B cells transfected with the hsa_circ_0001394 overexpression plasmid (pcDNA3.1) by qRT-PCR. The oligo probe was used to normalize hsa_circ_0001394 expression levels. **I** Relative levels of miR-527 in a sponge complex from hsa_circ_0001394 pull-down experiment with Hep3B lysates. qRT-PCR detected specific enrichment of miR-527 in the hsa_circ_0001394 probe group compared to the control probe group. **J** Negative correlation between the expression of hsa_circ_0001394 and miR-527 in HCC tissues and normal tissues by qRT-PCR. **K** Relative expression levels of miR-527 between HCC tissues and matched adjacent normal tissues. MiR-527 was downregulated in HCC tissues. All values are presented as mean ± SD from three independent experiments with similar results. (*p < 0.05, **p < 0.01, ***p < 0.001).

Previous studies have reported that circRNAs regulate target genes by sponging miRNAs to exert biological functions. To explore the downstream mechanism of hsa_circ_0001394, we used Circular RNA Interactome (https://circinteractome.nia.nih.gov/) to predict 6 miRNAs (context + score percentile ≥ 96), including miR-527 (99′), miR-518a-5p (99′), miR-370 (97′), miR-885-3p (97′), miR-657 (97′) and miR-510 (96′). MiR-527 and miR-518a-5p had the highest predicted scores (Fig. 3A), which potentially indicates these miRNAs absorbed by hsa_circ_0001394 in HCC cells. RNA pull-down assays were then performed with a specific biotinylated probe. The expression of the six potential miRNAs captured with this specific probe was significantly increased in HCC cells according to the fold changes determined using qRT-PCR. With the specific probe, we found that the hsa_circ_0001394 -miR-527 complex was notably enriched in HCC cells, while the other 5 miRNAs had no effective correlations with hsa_circ_0001394 (Fig. 3B). Hep3B and Huh7 cells were selected for further analysis because they exhibited the highest and lowest miR-527 expression, respectively, among the HCC cell lines according to qRT-PCR (Fig. 3C). Analysis with Kaplan–Meier Plotter (KM Plotter) showed that HCC patients with higher miR-527 expression tended to survive longer (http:// kmplot.com /analysis/index.php, *p* = 0.0098, Fig. 3D), indicating that miR-527 may function as a tumor suppressor corresponding to oncogenic hsa_circ_0001394. Based on these findings, we hypothesized that hsa_circ_0001394 may serve as a miR-527 sponge. To test this hypothesis, we investigated whether miR-527 expression is inversely related to hsa_circ_0001394 expression. Overexpression of hsa_circ_0001394 inhibited miR-527 expression in Hep3B cells, while knockdown of hsa_circ_0001394 resulted in a significant increase in miR-527 expression in Huh7 cells (Fig. 3E). In addition, to determine whether miR-527 directly binds to hsa_circ_0001394, we established a 3′-untranslated region (UTR) luciferase reporter assay including the WT and MUT fragments (Fig. 3F). After cotransfection of miR-NC or miR-527 mimics and WT or MUT hsa_circ_0001394 fragments, we observed significantly decreased luciferase activity in the WT fragment group but no obvious difference in the MUT group (Fig. 3G). A pull-down assay was conducted to further determine the interaction between hsa_circ_0001394 and miR-527 using the hsa_circ_0001394 probe. The biotinylated capture probe carrying a fluorescent barcode spans the back-splicing junction to ensure specificity. We first confirmed the significantly upregulated hsa_circ_0001394 probe pull-down efficiency in Hep3B cells transfected with hsa_circ_0001394 overexpression plasmids (pcDNA3.1). As is shown, the biotinylated hsa_circ_0001394 overexpression probe was successfully constructed. (Fig. 3H). The probes were incubated with C-1 magnetic beads to generate probe-coated beads, added to Hep3B cell lysates for overnight hybridization, and followed by washing away unbound RNAs. We then extracted RNA from probe-target mixtures and detected miR-527 expression levels in the sponge complexes using qRT-PCR. MiR-527 was significantly pulled down compared to the controls (Fig. 3I). Together, we confirmed that hsa_circ_0001394 interacted directly with miR-527. Correlation analysis showed that miR-527 expression was negatively correlated with hsa_circ_0001394 expression (Fig. 3J), and miR-527 expression was significantly decreased in HCC specimens according to qRT-PCR (Fig. 3K).

**Fig. 4:**
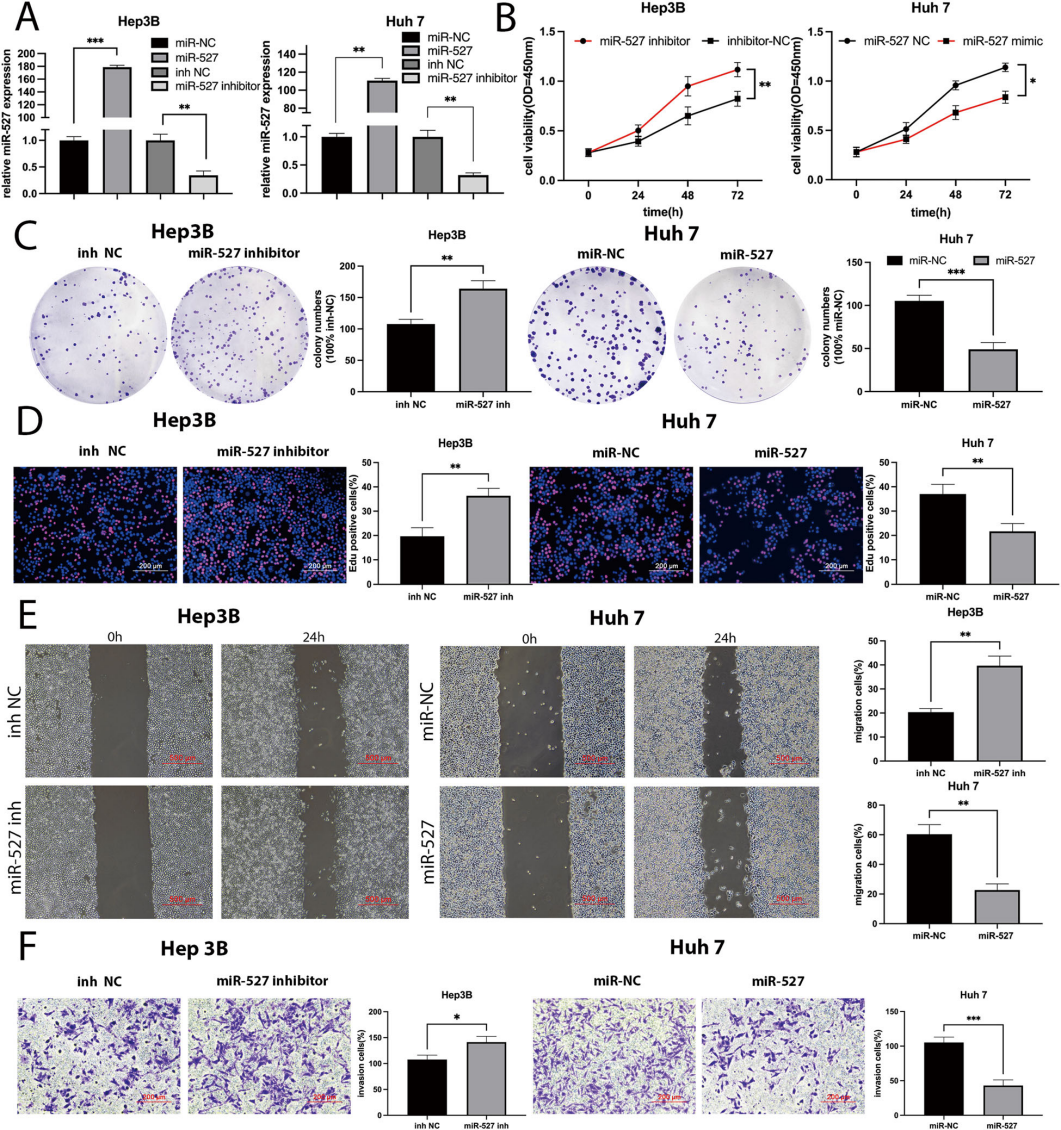
MiR-527 inhibits HCC progression. **A** Relative miR-527 expression of HCC cells transfected with miR-527 mimics or corresponding negative controls (miR-NC) and miR-527 inhibitors or corresponding negative controls (inh-NC) according to qRT-PCR. **B** MiR-527 significantly inhibited the cell growth while miR-527 inhibitors promoted HCC cell growth as determined by CCK-8 assays. **C** MiR-527 significantly inhibited the colony formation, while miR-527 inhibitors exerted the opposite effect on colony formation. **D** EdU assays showed that miR-527 significantly suppressed DNA synthesis, while miR-527 inhibitors exerted the opposite effect on the HCC cell DNA synthesis. **E** Wound-healing assays were used to compare the migration abilities of Hep3B and Huh7 cells transfected with inh-NC or miR-527 inhibitors and miR-NC or miR-527 mimics. **F** The effect of miR-527 on HCC cell invasion abilities was determined by Transwell assays. MiR-527, miR-527 mimics for miR-527 overexpression system; miR-NC, negative control plasmids relative to miR-527 mimics; miR-527 inh, miR-527 inhibitors; inh-NC, relatively control plasmids of miR-527 inhibitors. All values are presented as mean ± SD from three independent experiments with similar results. (*p < 0.05, **p < 0.01, ***p < 0.001).

To further investigate the detailed antioncogenic role of miR-527 revealed by KM Plotter (Fig. 3D), functional experiments were conducted. The efficiency of transfection with miR-NC, miR-527 mimics, inh-NC, and miR-527 inhibitors were evaluated by qRT-PCR (Fig. 4A). The results revealed that miR-527 overexpression inhibited cell viability, while knockdown of miR-527 markedly increased cell viability (Fig. 4B). The colony number of HCC cells transfected with miR-527 inhibitors was much greater than that of controls, whereas the opposite result was obtained for HCC cells transfected with miR-527 mimics (Fig. 4C). Similarly, downregulation of miR-527 increased the percentage of EdU-positive cells, while upregulation of miR-527 decreased the percentage of Edu-positive cells (Fig. 4D). Subsequently, the influence of miR-527 on migration and invasion was assessed by wound-healing and Transwell assays. Migration ability was significantly promoted in the miR-527-knockdown group, but this effect was reversed by miR-527 treatment (Fig. 4E). Regarding invasion ability, we observed similar tendencies in the same groups in the Transwell assay (Fig. 4F). In conclusion, these results demonstrated that miR-527 is downregulated in HCC tissues and acts as a tumor suppressor by reversing the promoting effects of hsa_circ_0001394.

### UBE2A acts as a direct target of miR-527

**Fig. 5:**
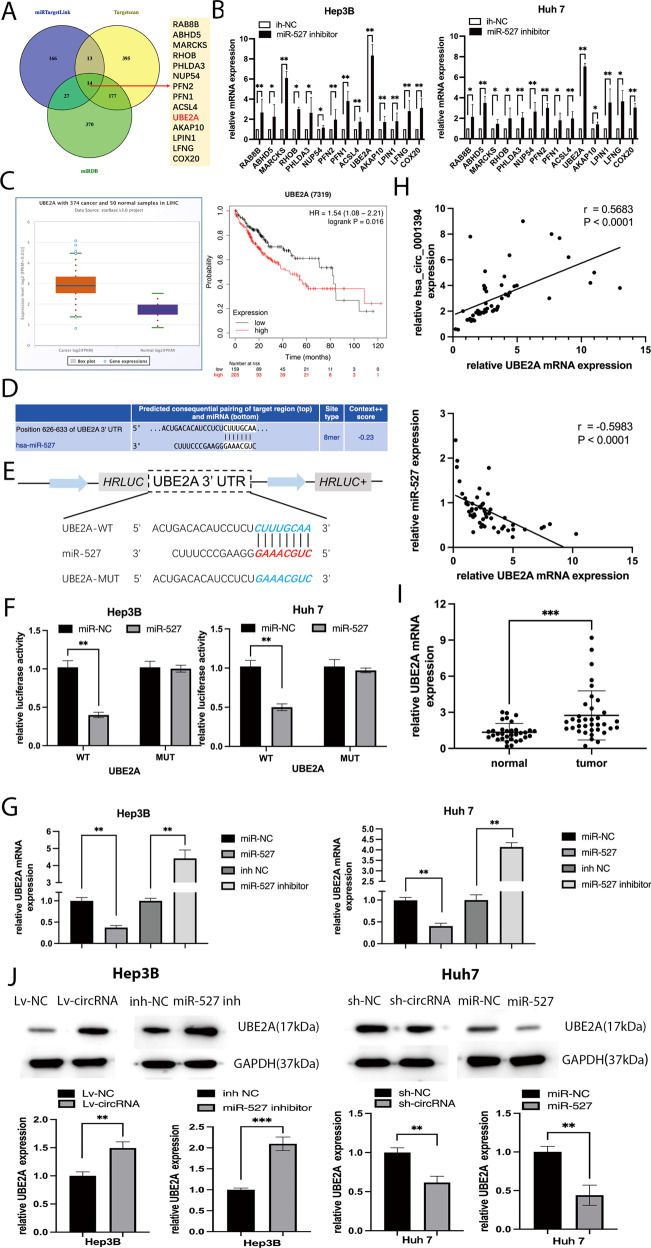
UBE2A acts as a direct target of miR-527. **A** TargetScan, miRTargetLink, and miRDB were used to predict the potential target mRNAs of miR-527. **B** Relative mRNA expression in Hep3B and Huh7 cells transfected with inh-NC or miR-527 inhibitors and miR-NC or miR-527 mimics as determined by qRT-PCR. The histogram shows relative mRNA levels. **C** The expression levels of UBE2A in 374 HCC tissues and 50 normal samples as well as the relationship between survival possibility and the expression of UBE2A in HCC obtained from TGGA database. UBE2A was significantly upregulated in HCC tissues, and patients with high UBE2A expression had a lower overall survival based on the analysis of TGGA database. **D** Potential binding sites between miR-527 and the 3′-UTR of UBE2A as analyzed by TargetScan. **E** Schematic illustration of the complementary sequences between miR-527 and UBE2A 3′-UTR WT (wild-type) or MUT (mutated-type). **F** Relative luciferase activities were used to detect the binding sites after cotransfection of miR-NC, miR-527 mimics, UBE2A-MUT, and UBE2A-WT into Hep3B and Huh7 cells. Cotransfection of miR-527 mimics and UBE2A-WT significantly decreased the luciferase activity, while the luciferase activity after cotransfection of miR-527 mimics and UBE2A-MUT showed no obvious change. **G** Relative UBE2A levels of HCC cell lines transfected with miR-NC, miR-527 mimics, inh-NC, and miR-527 inhibitors according to qRT-PCR. UBE2A was downregulated after transfection with miR-527 mimics, while transfection with miR-527 inhibitors resulted in upregulation of UBE2A. **H** Correlation analysis between the expression of UBE2A and hsa_circ_0001394, as well as between UBE2A and miR-527. Hsa_circ_0001394 was positively correlated with UBE2A while miR-527 was negatively correlated with UBE2A. **I** Relative UBE2A mRNA expression levels between HCC and matched normal tissues according to qRT-PCR. UBE2A was upregulated in HCC tissues. **J** Evaluation of UBE2A protein levels by western blotting in HCC cells transfected with Lv-NC, Lv-circ, sh-NC, sh-circ, miR-NC, miR-527 mimics, inh-NC, and miR-527 inhibitors. UBE2A protein levels were enhanced after transfection with Lv-circRNA but reduced after transfection with sh-circRNA. However, UBE2A protein levels decreased after transfection with miR-527 mimics but were enhanced after transfection with miR-527 inhibitors. All values are presented as mean ± SD from three independent experiments with similar results. (*p < 0.05, **p < 0.01, ***p < 0.001).

To better verify the mechanism of miR-527, bioinformatics databases including TargetScan (https://www.targetscan.org/vet.72/), miRDB (https://mirdb.org/), and miRTarget Link 2.0 (https://ccb-compute.cs.uni-saarland.de/mirtargetlink2) were used to search for potential downstream target genes, and 14 genes were selected (Fig. 5A). We detected the expression levels of these 14 mRNAs in miR-527-knockdown HCC cells, and among the 14 upregulated genes identified in this manner, only ubiquitin-conjugating enzyme E2A (UBE2A) expression was most prominently upregulated (Fig. 5B), suggesting that UBE2A expression may be most likely affected by miR-527. UBE2A was determined to be expressed at higher levels in HCC specimens than in normal tissues with GEPIA (https://gepia.cancer-pku.cn/), and patients with lower UBE2A expression had longer survival according to KM Plotter (Fig. 5C). Interestingly, it has been previously reported that upregulation of UBE2A predicts poor HCC prognosis [33]. Subsequently, according to potential binding sites between the UBE2A UTR and miR-527 (Fig. 5D), we constructed UBE2A MUT and WT sequences (Fig. 5E). As expected, the luciferase activity of the UBE2A-WT group was markedly lower than that of the control group; in contrast, the mutant group showed no obvious decrease (Fig. 5F). Moreover, the transfection efficiency of miR-527 knockdown and overexpression in HCC cell lines was evaluated by qRT-PCR (Fig. 5G). Correlation analysis showed that UBE2A expression was positively correlated with hsa_circ_0001394 expression but negatively correlated with miR-527 expression (Fig. 5H). As detected by qRT-PCR, UBE2A was highly expressed, in 33 pairs of HCC tissues and matched normal tissues (Fig. 5I). Furthermore, western blotting demonstrated that UBE2A protein levels were enhanced after transfection with Lv-circRNA but reduced after transfection with sh-circRNA. However, miR-527 played a completely different role. Upregulation of miR-527 strongly inhibited UBE2A protein expression, while downregulation had the opposite effect (Fig. 5J). These results suggested that UBE2A plays an oncogenic role as a direct target of miR-527 but is indirectly regulated by hsa_circ_0001394.

### Hsa_circ_0001394 regulates UBE2A by sponging miR-527 through the MDM2 /p53/ Bcl2/Bax pathway

**Fig. 6:**
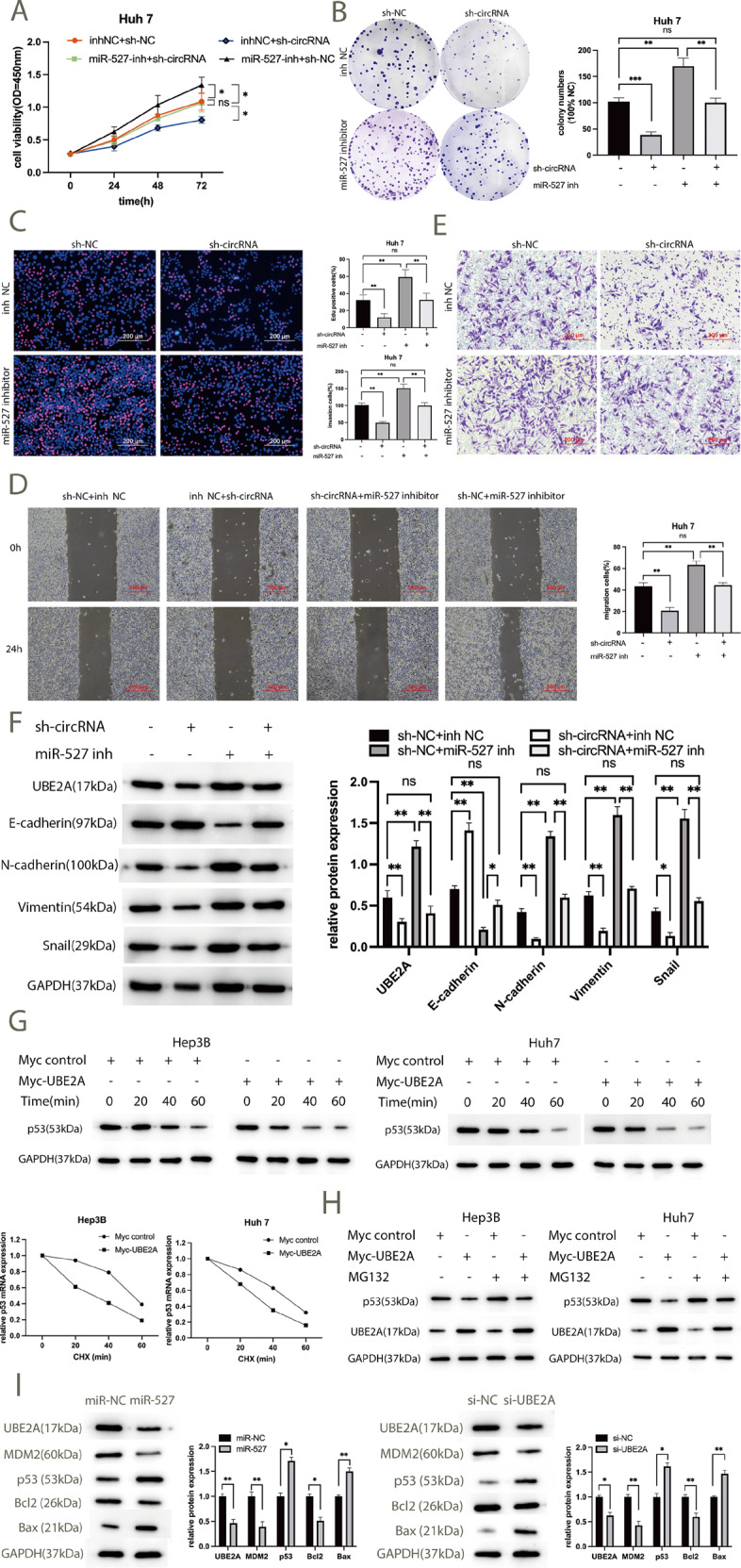
Hsa_circ_0001394 regulates UBE2A by sponging miR-527. Sh-NC, sh-circ, inh-NC, and miR-527 inhibitors have been cotransfected into HCC cells. **A** CCK-8 assays showed that downregulation of miR-527 abolished the inhibitory effect of hsa_circ_0001394 knockdown on Huh7 cell proliferation. **B** Downregulation of miR-527 alleviated the suppression of colony formation induced by hsa_circ_0001394 knockdown in Huh7 cells. **C** Knocking down miR-527 reversed the inhibitory effect of the EdU assay achieved by knocking down hsa_circ_0001394. **D** Silencing of hsa_circ_0001394 caused a decrease in migration abilities, which was reversed by miR-527 inhibitors. **E** The reduction in the invasion abilities of Huh7 cells mediated by hsa_circ_0001394 silencing was successfully attenuated by miR-527 inhibitors. **F** Relative protein expression levels of UBE2A and EMT-related proteins, including E-cadherin, N-cadherin, vimentin, and snail, according to western blotting. GAPDH was used as an internal control. The protein levels of N-cadherin, vimentin, and snail were lower in the hsa_circ_0001394-deficient group, while the expression of E-cadherin exerted the opposite result. MiR-527 inhibitors attenuated the upregulation of E-cadherin and downregulation of UBE2A, N-cadherin, vimentin, and snail levels induced by hsa_circ_0001394 deficiency. **G** Overexpression of UBE2A decreased the half-life of p53 in Hep3B and Huh7 cells. HCC cells were transfected with myc control or myc-UBE2A plasmids followed by treatment with cycloheximide (CHX) for the indicated periods of time. The protein expression of UBE2A and p53 was examined using western blot analysis. **H** After transfection with myc control or myc-UBE2A plasmids, HCC cells were treated with MG132 for 6 h and subjected to western blot analysis. UBE2A upregulation resulted in a significant decrease in p53 protein levels, which was blocked by MG132. **I** The protein expression of UBE2A, MDM2, Bcl2, p53, and Bax was determined using western blot analysis in Huh7 cells transfected with miR-NC or miR-527 mimics and si-NC or si-UBE2A (silencing of UBE2A). GAPDH was used as an internal control. Compared to corresponding control groups, transfection of Huh7 cells with miR-527 mimics or with si-UBE2A decreased MDM2 and Bcl2 levels but increased p53 and Bax levels. Myc-UBE2A, UBE2A overexpression system construction with CMV-Myc plasmids expressing UBE2A; Myc control, relative control plasmids of myc-UBE2A; si-UBE2A, small interfering RNAs (siRNAs) against UBE2A; si-NC, relative control plasmids of si-UBE2A. All values are presented as mean ± SD from three independent experiments with similar results. (*p < 0.05, **p < 0.01, ***p < 0.001).

Rescue experiments were performed to further clarify the effects of hsa_circ_0001394 and miR-527 on HCC progression. First, sh-NC or sh-circRNA and inh-NC or miR-527 inhibitor were cotransfected into HCC cells. A CCK-8 assay was then performed, which revealed that the miR-527 inhibitor notably blocked the effect of hsa_circ_0001394 knockdown group on cell viability (Fig. 6A). Similarly, cell colony numbers were suppressed by silencing of hsa_circ_0001394, but treatment with the miR-527 inhibitor reversed this effect. (Fig. 6B). Knockdown of hsa_circ_0001394 significantly decreased the percentage of EdU-positive cells. After transfection with the miR-527 inhibitor, the percentage was increased (Fig. 6C). Furthermore, wound-healing and Transwell assays demonstrated that migration and invasion abilities were inhibited by silencing hsa_circ_0001394, but miR-527 inhibitor treatment attenuated these effects (Fig. 6D, E). Western blotting assays also showed that downregulation of UBE2A protein expression via knockdown of hsa_circ_0001394 was attenuated after transfection with the miR-527 inhibitor. Next, we investigated epithelial–mesenchymal transition (EMT)-related biomarkers, including E-cadherin, N-cadherin, vimentin, and snail. Knockdown of hsa_circ_0001394 resulted in increased E-cadherin expression but decreased N-cadherin, vimentin, and snail expression, which could be reversed by miR-527 inhibitor treatment. (Fig. 6F). It has been reported that UBE2A could regulate p53 protein levels by transcription and post-transcription mechanisms [33]. To further explore the effect of UBE2A on p53 degradation, a cycloheximide (CHX) chase experiment was conducted. After transfection with UBE2A overexpression plasmids (Myc-UBE2A) or control plasmids (Myc), Hep3B and Huh7 cells were treated with 50 μg/ml CHX for every 20 min, lysed and subjected to western blotting. We found that upregulation of UBE2A resulted in a decrease in the half-life of p53 and markedly promoted p53 degradation, as compared to the control group (Fig. 6G). Previous studies have demonstrated that UBE2A serving as an E2 ligase promotes the ubiquitination and degradation of p53 protein levels by forming a ternary complex with MDM2 and p53 [34]. MG132, an inhibitor of E3 ligases, can inhibit ubiquitination and 26 S proteasome-mediated degradation. After transfection with Myc-UBE2A or Myc control plasmids, Hep3B and Huh7 cells were treated with MG132 for 6 h and followed by western blotting. Consistently, overexpression of UBE2A resulted in a significant decrease in p53 protein levels, which was abrogated by MG132 (Fig. 6H). P53, releasing from its upstream negative regulator MDM2, inhibits cell proliferation and induces cell apoptosis via a decreased Bcl2/Bax ratio [35]. To investigate the potential mechanism of UBE2A in HCC, MDM2, p53, Bcl2, and Bax protein levels in Huh7 cells were determined by western blotting. Compared to the negative control group (miR-NC), upregulation of miR-527 reduced MDM2 and Bcl2 expression but enhanced p53 and Bax protein levels. Moreover, MDM2 and Bcl2 expression decreased in UBE2A-silenced (si-UBE2A) group, while p53 and Bax expression increased relative to that in the corresponding control (si-NC) group (Fig. 6I). Overall, the effects of hsa_circ_0001394 on HCC proliferation, migration, and invasion are counteracted by miR-527 and may be related to EMT signaling. UBE2A mediates HCC malignant behaviors by promoting tumor suppressor p53 degradation and interacting with miR-527 through the MDM2/p53/Bcl2/Bax pathway.

### Silencing of hsa_circ_0001394 inhibits HCC tumorigenesis in vivo

**Fig. 7:**
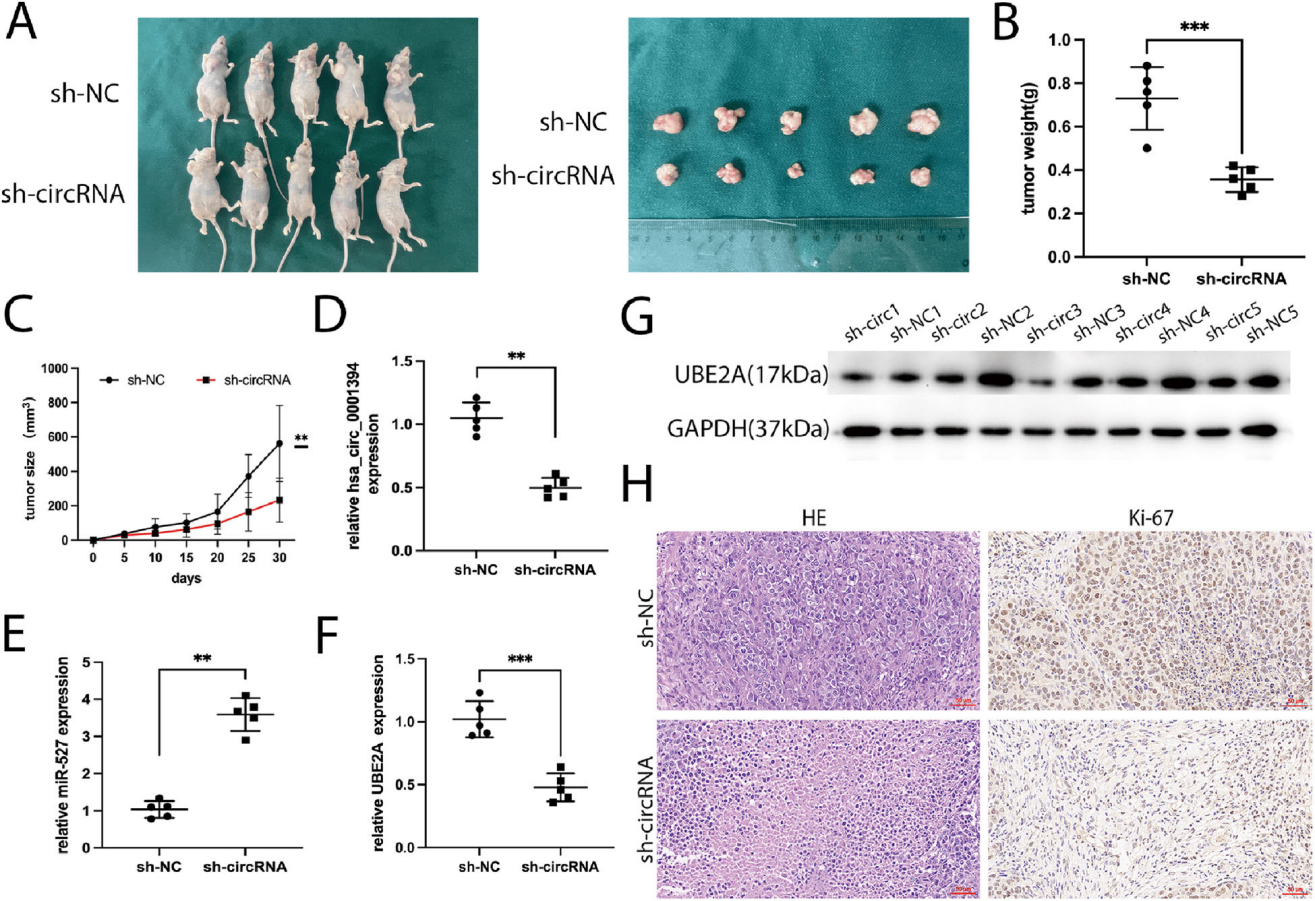
Knockdown of hsa_circ_0001394 suppresses HCC progression in vivo. **A** Five pairs of nude mice were subcutaneously injected with Huh7 cells transfected with sh-NC or sh-circRNA plasmids. After 30 days, HCC tumors were dissected and photographed. **B** Tumor weight was calculated on the day mice were euthanized. The tumor weight of hsa_circ_0001394-deficient group was markedly lower than that of the control group. **C** Tumor volumes were recorded every 5 days starting on the day when mice were inoculated with Huh7 cells transfected with sh-NC or sh-circRNA. Hsa_circ_0001394 knockdown decreased tumor volumes relative to the control group. **D** Hsa_circ_0001394 was downregulated in the hsa_circ_0001394-deficient group relative to the control group according to qRT-PCR. **E** MiR-527 was upregulated in the sh-circRNA group compared to the control group according to qRT-PCR. **F** UBE2A was significantly expressed at lower levels in the hsa_circ_0001394 knockdown group than in the control group as determined by qRT-PCR. **G** The protein levels of UBE2A were compared between the sh-NC nude mouse models and sh-circRNA models using western blot analysis. Hsa_circ_0001394 silencing reduced the protein levels of UBE2A. **H** Representative images of hematoxylin–eosin (HE) staining of sections of xenograft tumors formed by Huh7 cells. Ki-67 staining of tumors was determined by immunohistochemical analysis. All values are presented as mean ± SD from three independent experiments with similar results. (*p < 0.05, **p < 0.01, ***p < 0.001).

To further investigate the function of hsa_circ_0001394 in HCC tumorigenesis in vivo, five pairs of xenograft models were established by subcutaneously injecting Huh7 cells transfected with sh-circRNA or sh-NC plasmids into BALB/c nude mice. The tumors were excised after 30 days (Fig. 7A). The weight of the tumors that formed in the hsa_circ_0001394-deficient group was significantly lower than that in the negative control group (Fig. 7B). The growth of tumors after inoculation with sh-circRNA-transfected Huh7 cells was much slower than that after inoculation with control cells (Fig. 7C). qRT-PCR was utilized to determine the expression of hsa_circ_0001394, miR-527, and UBE2A. Hsa_circ_0001394 silencing (Fig. 7D) resulted in upregulation of miR-527 (Fig. 7E) but downregulation of UBE2A mRNA (Fig. 7F). The UBE2A protein levels tended to be lower in sh-circRNA models than in control models (Fig. 7G). Furthermore, HE staining and IHC assays were conducted. According to the staining, Ki-67 was weakly expressed in hsa_circ_0001394-deficient silenced models (Fig. 7H). Taken together, these findings indicated that knockdown of hsa_circ_0001394 inhibits HCC tumorigenesis in vivo.

## Discussion

The advancement of RNA-seq technology and bioinformatics tools has enabled the discovery of increasing numbers of RNA transcripts [9]. CircRNAs, which are composed of exonic or intronic sequences, are novel covalent, conserved, tissue-specific, and abundant RNAs that are found mainly in the cytoplasm [36]. Additionally, emerging evidence demonstrates the involvement of circRNAs in various diseases, especially cancers [37]. Previous studies have elucidated the differential expression and functions of circRNAs in HCC; however, the downstream molecular mechanisms remain unclear [38]. CircRNAs are stable and abundant in body fluids, including plasma, urine, and exosome fluid, indicating that they have potential value for tumor liquid biopsies [39]. In the present study, we identified a novel circular RNA, hsa_circ_0001394, by RNA-seq analysis and qRT-PCR between HCC tissues and matched adjacent tissues, and we demonstrated that hsa_circ_0001394 played a regulatory role in HCC progression. Subsequently, we found that hsa_circ_0001394 was upregulated in HCC cell lines, tissues, and plasma. Clinicopathological assessment illustrated that hsa_circ_0001394 expression was significantly correlated with tumor differentiation, vascular invasion, and AFP levels, suggesting that hsa_circ_0001394 expression may be correlated with HCC prognosis and might function as a noninvasive biomarker for the early detection and prognosis prediction of HCC in patients. Moreover, functional experiments were conducted to confirm that hsa_circ_0001394 facilitated the proliferation, migration, and invasion of HCC cells. Knockdown of hsa_circ_0001394 inhibited HCC tumorigenesis in vivo.

We then employed bioinformatics tools to determine the downstream mechanism by which hsa_circ_0001394 exerts its biological effects in HCC. MiR-527 was identified as the target of hsa_circ_0001394. Previous studies have revealed that circRNAs can function as sponges of miRNAs. Interestingly, recent research has indicated the antioncogenic role of miR-527 in various cancers. For example, miR-527 has been reported to be downregulated in gastric cancer and to inhibit cell proliferation, migration, and EMT by regulating Sp1 [40]. Moreover, miR-527 has been shown to inhibit lung cancer progression by interacting with BRF2 [41]. By sponging the antioncogene miR-527, circ-CDC45 facilitates glioma cell progression [42]. Another circRNA, hsa_circ_0001495, increases Robo1 expression by targeting miR-527 to promote malignant behaviors of bladder cancer cells [43]. However, few studies have explored the role of miR-527 in HCC. Our findings were consistent with an antioncogenic role of miR-527. We investigated whether miR-527 countervails the oncogenic role of hsa_circ_0001394 as a suppressive factor inhibiting HCC proliferation, migration, and invasion. Existing evidence indicates that competing endogenous RNA (ceRNA) transcripts regulate each other by competing with shared miRNAs. CircRNAs are recognized to act as important regulators of protein expression by sponging miRNAs.

**Fig. 8:**
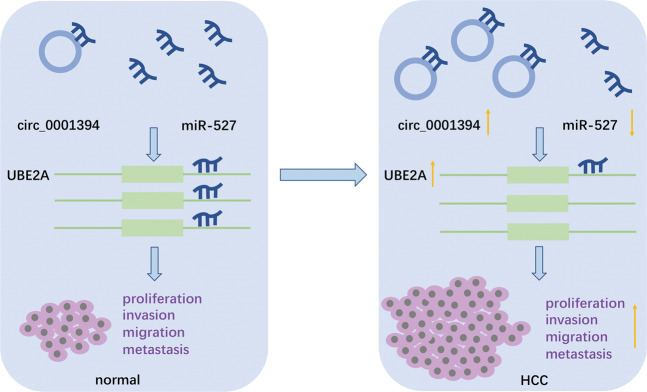
The hsa_circ_0001394/miR-527/UBE2A axis promotes HCC proliferation, migration, and invasion. HCC hepatocellular carcinoma.

Ubiquitin-conjugating enzyme E2A (UBE2A), which contains a 456-bp open reading frame, was predicted as a downstream target of miR-527 using bioinformatics tools, luciferase activity assays, qRT-PCR, and western blotting. It has been reported that increased UBE2A expression predicts poor survival in patients with ovarian cancer [44]. In addition, an RNA-seq signature involving UBE2A can predict the prognosis of multiple myeloma (MM) [45]. UBE2A has also been found to be an important contributor to tumorigenesis [46]. Notably, one study has demonstrated that upregulation of UBE2A predicts poor prognosis in HCC [33], corresponding to our findings. The ubiquitin system plays a vital role in various biological processes including DNA repair, gene expression, and cell cycle modifications. Protein ubiquitination requires the ubiquitin-activating enzyme E1, ubiquitin-conjugating enzyme E2 (or ubiquitin carrier protein), and ubiquitin-protein ligase (E3) [47]. UBE2A belongs to a group of E2 enzymes. Previous studies have shown that UBE2A promotes p53 degradation through the ubiquitin-proteasome pathway [34]. P53, a tumor suppressor, has been found to participate in various types of tumorigenesis and to be associated with cell proliferation, apoptosis, DNA repair, and cell cycle arrest [48]. Murine double minute 2 (MDM2), an upstream regulator of p53, plays vital role in promoting tumorigenic processes by inhibiting p53 expression [49]. Moreover, p53 ubiquitination by UBE2A requires the involvement of MDM2 by forming a ternary complex [34]. B-cell lymphoma-2 (Bcl2) family members have been recognized as targets in cell growth arrest or apoptosis mediated by p53. Bcl2 plays an antiapoptotic role by antagonizing the biological effect of p53, while Bcl2-associated X protein (Bax) and Bak serve as pro-apoptotic proteins [50]. Releasing from its negative upstream regulator (i.e., MDM2), p53 inhibits Bcl2 but enhances Bax expression, thereby impairing cell proliferation and inducing apoptosis. Here, we report, for the first time, the role of the hsa_circ_0001394/miR-527/UBE2A regulatory axis in HCC progression (Fig. 8). Hsa_circ_0001394 sponges miR-527 to modify UBE2A expression. Collectively, hsa_circ_0001394 and UBE2A serve as tumor promotors while miR-527 acts as a suppressor in HCC progression. High expression of hsa_circ_0001394 results in miR-527 deficiency and subsequent UBE2A upregulation, which promotes HCC malignant behaviors by inhibiting p53 by enhancing the levels of MDM2 and Bcl2 but suppressing Bax protein levels. The hsa_circ_0001394/miR-527/UBE2A regulatory axis provides a perspective on the development of HCC.

Notably, there were several limitations of this study. First, more HCC specimens and plasma samples should be analyzed to confirm the results. In addition, whether other miRNAs or target genes that interact with circRNAs participate in HCC progression remains to be investigated. Finally, the downstream pathway of UBE2A needs to be further explored to clarify the mechanism of HCC progression. Therefore, further study will be important to convincingly establish hsa_circ_0001394 as a noninvasive biomarker and to acquire a more thorough understanding of the detailed molecular mechanism of HCC.

## Supplementary information

Original Data File

## Data Availability

The datasets used or analyzed during this study are available from the corresponding authors on reasonable request.
