# Correction to: The circular RNA hsa_circ_0001394 promotes hepatocellular carcinoma progression by targeting the miR-527/UBE2A axis

**DOI:** 10.1038/s41420-022-00955-0

**Published:** 2022-03-31

**Authors:** Yu Yan, Yu Nie, Chun Peng, Fuchen Xing, Saiguang Ji, Hong Liu, Chuandong Zhu

**Affiliations:** 1 Department of Oncology, The Second Hospital of Nanjing, Nanjing University of Chinese Medicine, Nanjing, 210003 China; 2 Department of Thoracic Surgery, The Second Hospital of Nanjing, Nanjing University of Chinese Medicine, Nanjing, 210003 China

**Keywords:** Tumour biomarkers, Targeted therapies, Tumour biomarkers

Correction to: *Cell Death Discovery* 10.1038/s41420-022-00866-0, published online 24 February 2022

The original version of this article contained a mistake. The equal contribution statement should be deleted “These authors contributed equally: Yu Yan, Yu Nie, Chun Peng, Fuchen Xing and Saiguang Ji”. The original article has been corrected.

