# Supplementary material for: The circular RNA hsa_circ_0001394 promotes hepatocellular carcinoma progression by targeting the miR-527/UBE2A axis
Source: Cell Death Discov. 2022 Feb 24;8:81. doi: 10.1038/s41420-022-00866-0 (PMC8873434; doi:10.1038/s41420-022-00866-0)

Fig.5J

Hep3B

GAPDH (37kDa)

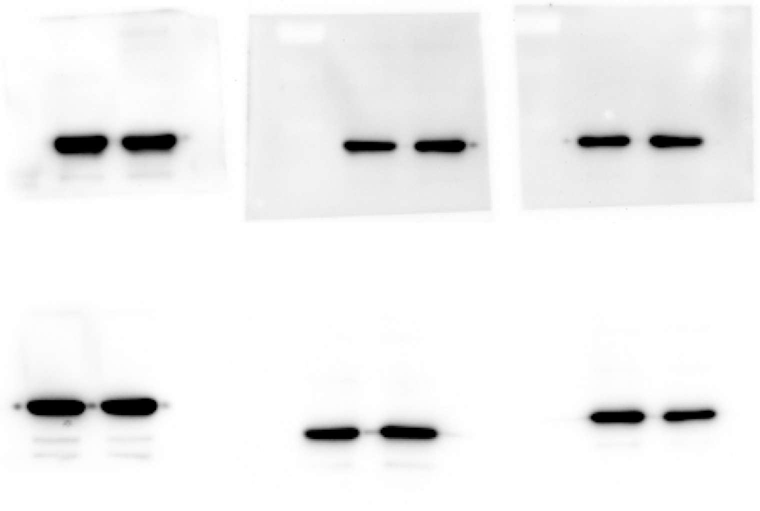

UBE2A (17kDa)

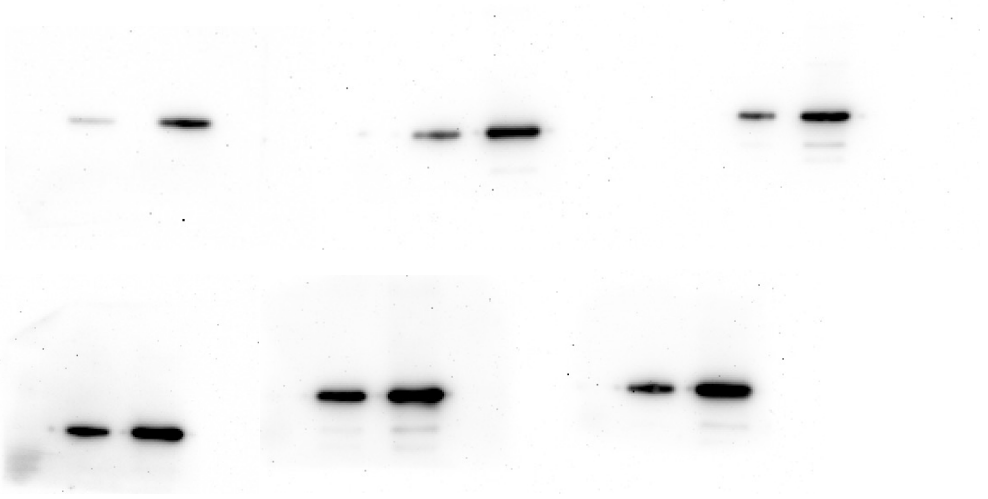

Huh7

GAPDH (37kDa)

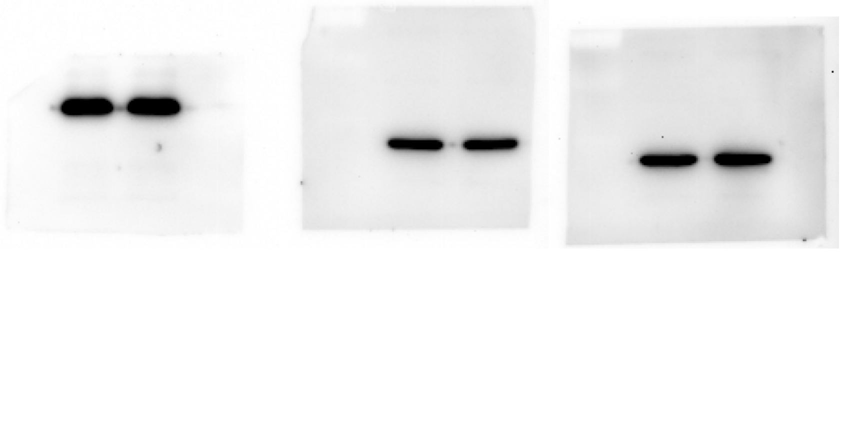

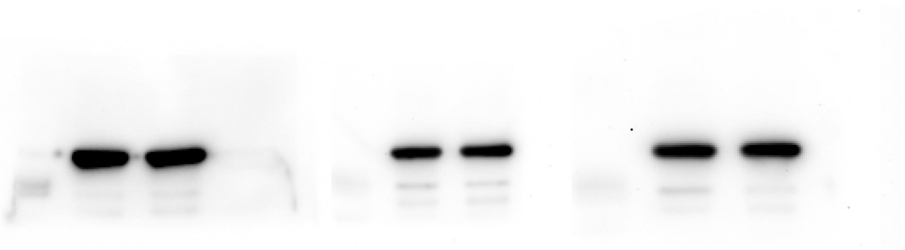

UBE2A (17kDa)

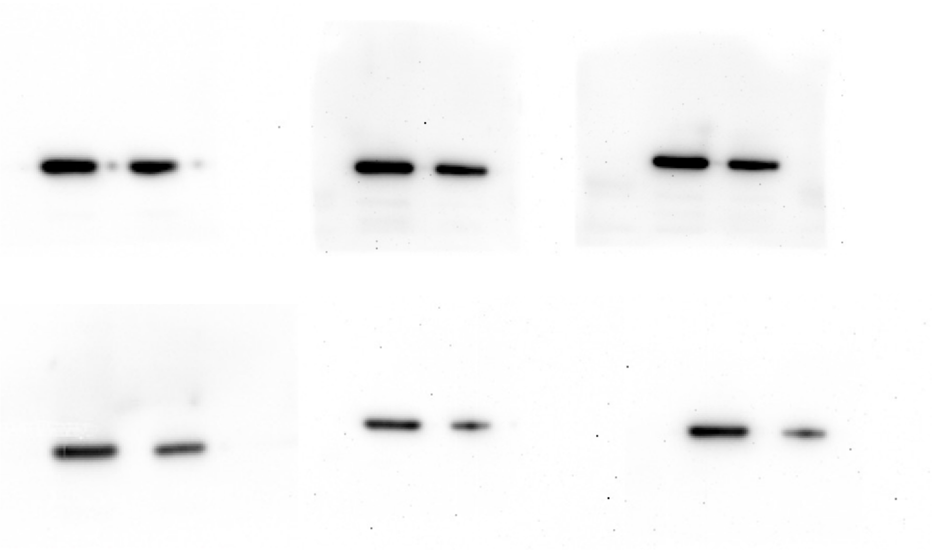

Fig6.F
UBE2A (17kDa)

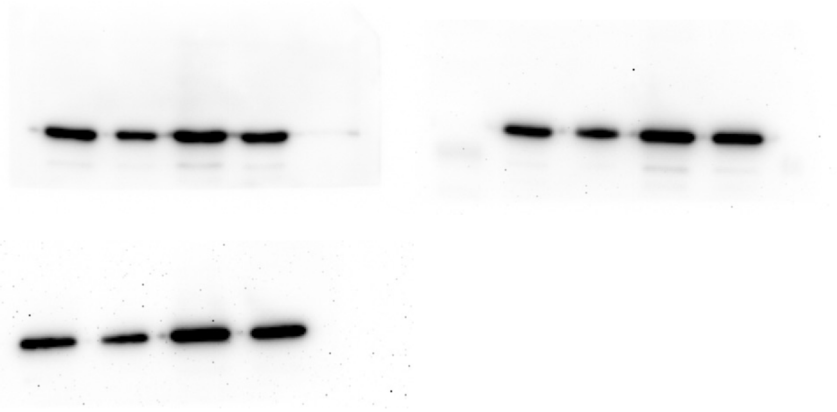

E-cadherin (97kDa)

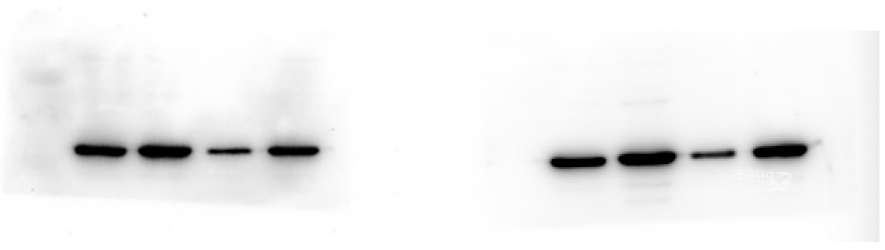

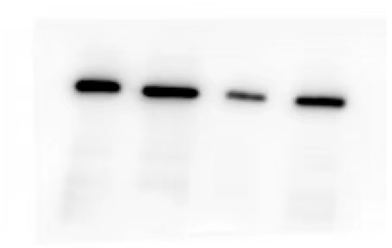

N-cadherin (100kDa)

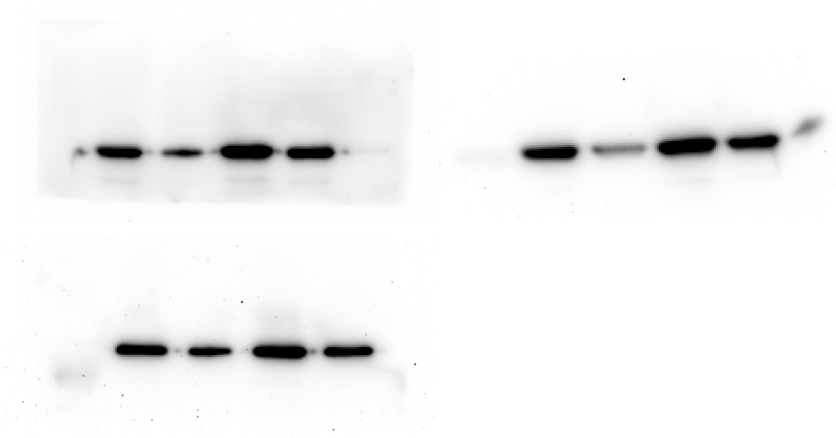

Vimentin (54kDa)

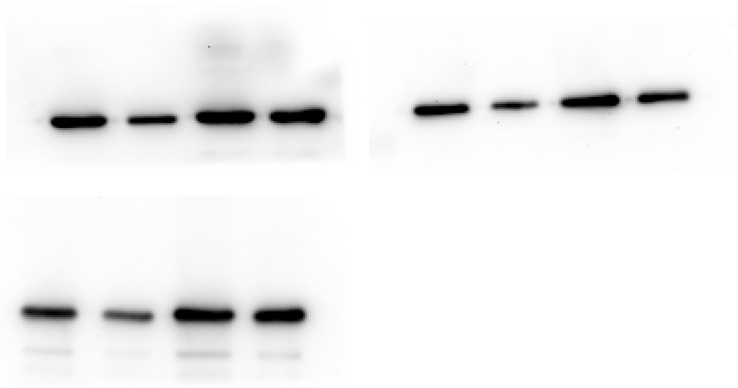

Snail (29kDa)

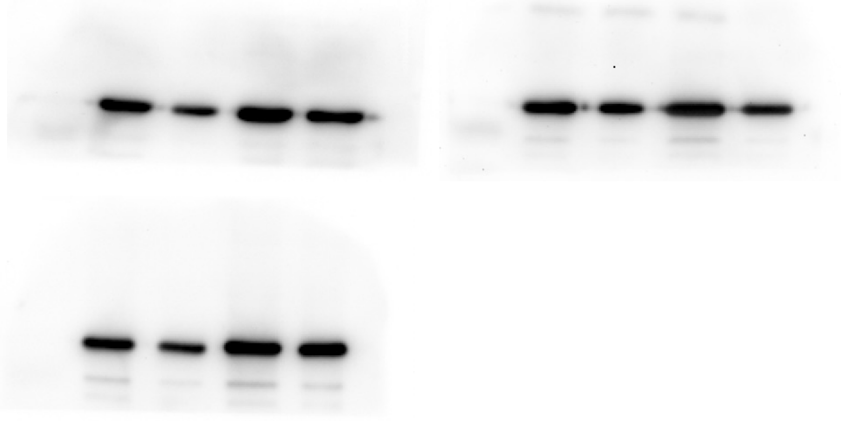

GAPDH (37kDa)

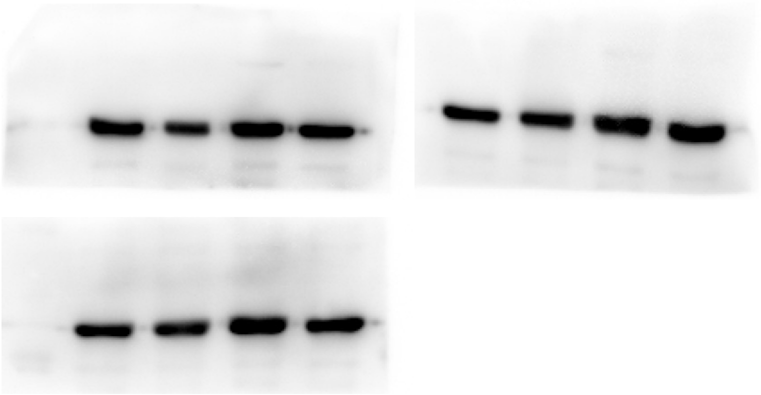

Fig6.G

Hep3B
p53 (53kDa)

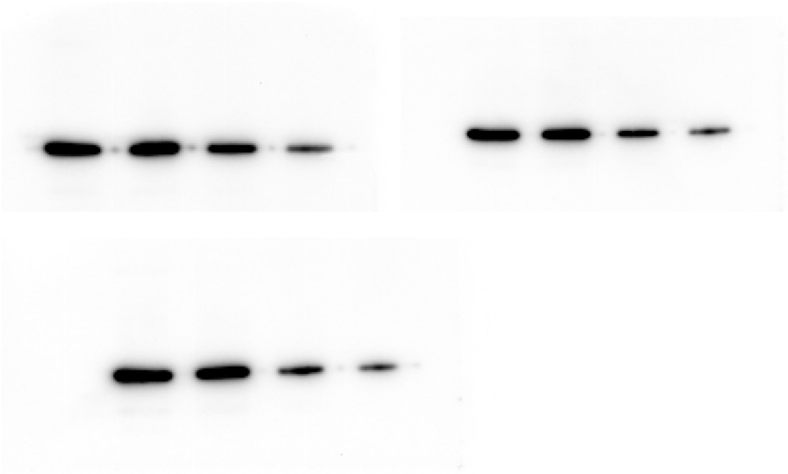

GAPDH (37kDa)

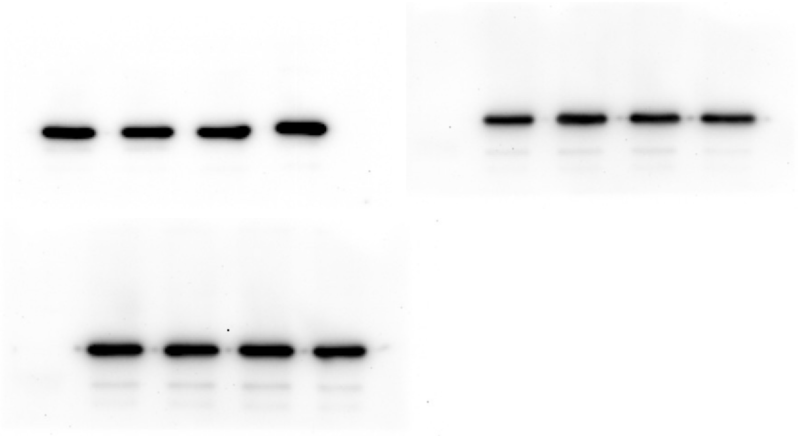

p53 (53kDa)

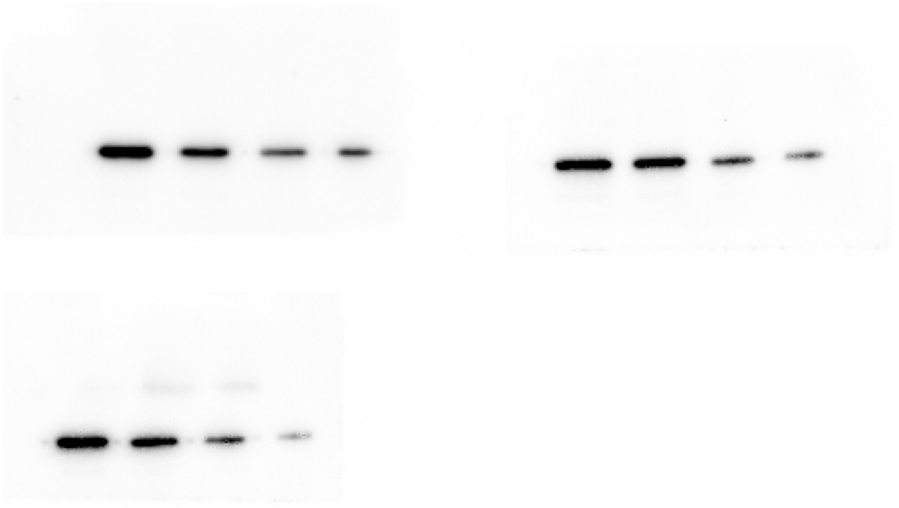

GAPDH (37kDa)

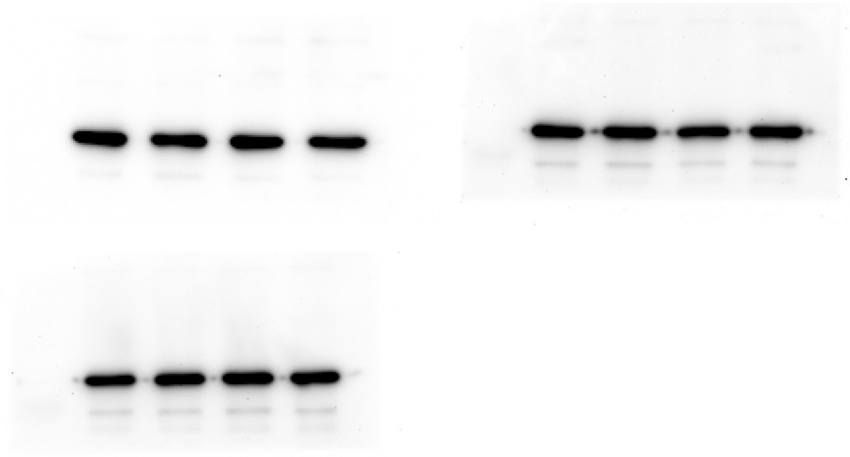

Huh7
p53 (53kDa)

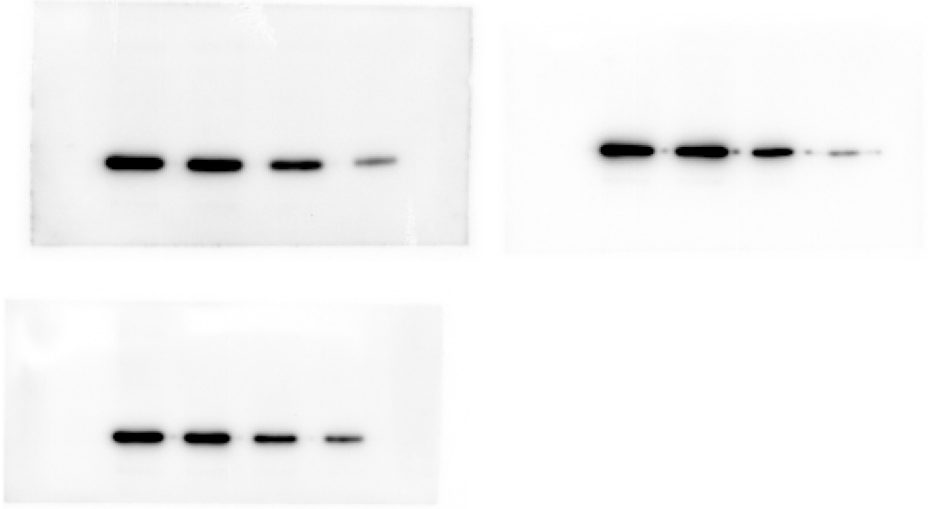

GAPDH (37kDa)

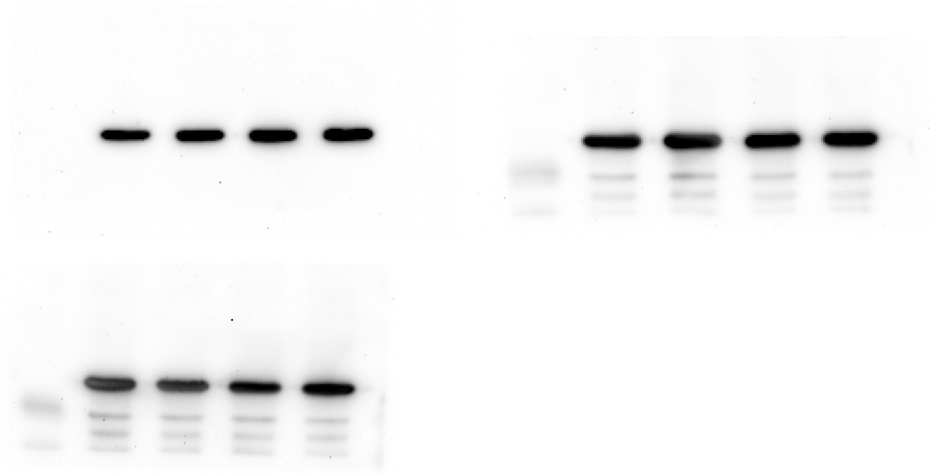

p53 (53kDa)

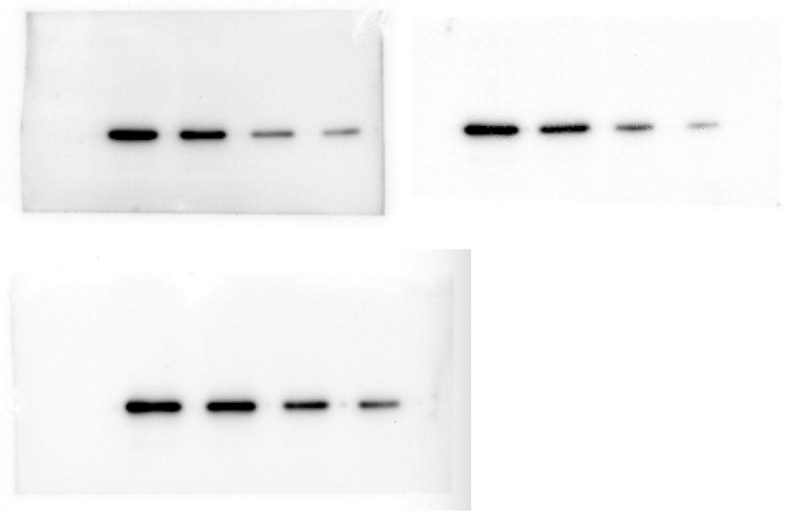

GAPDH (37kDa)

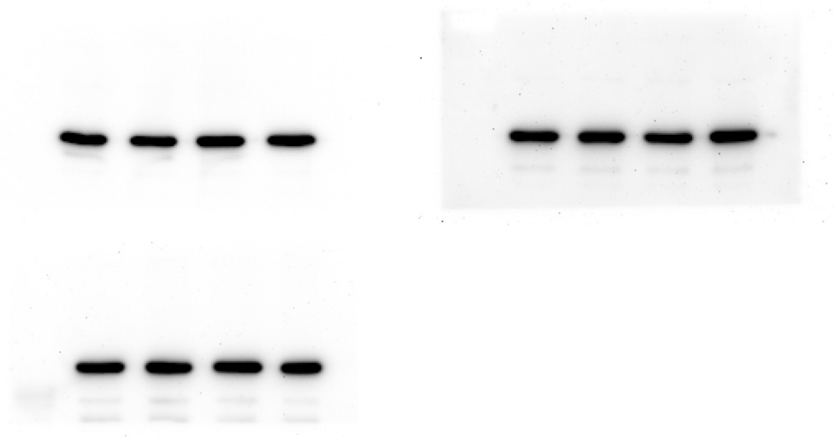

Fig6.H

Hep3B
P53 (53kDa)

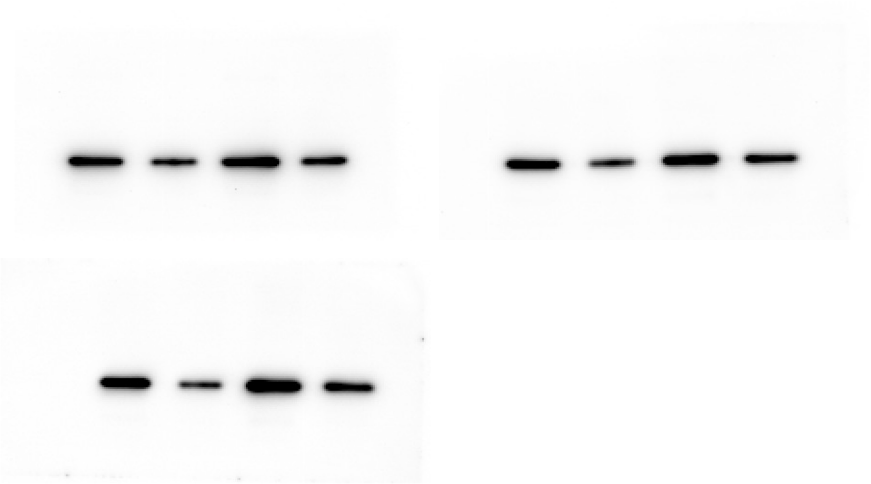

UBE2A (17kDa)

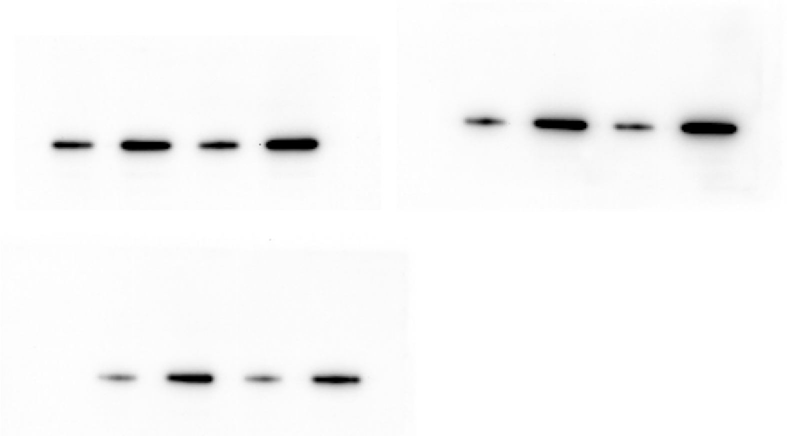

GAPDH (37kDa)

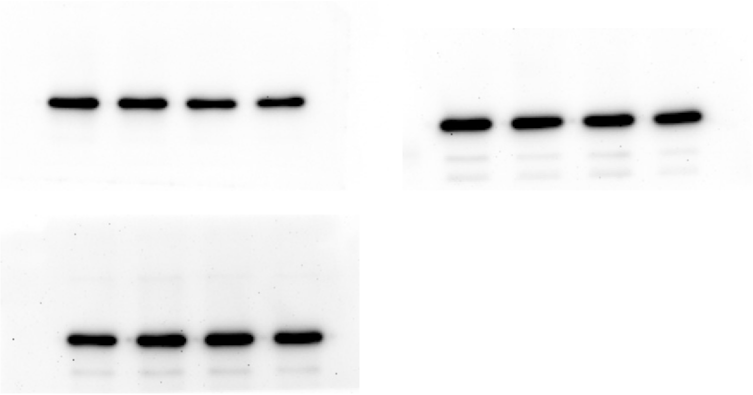

Huh7
P53 (53kDa)

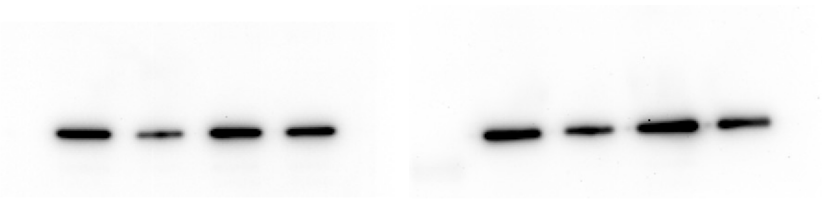

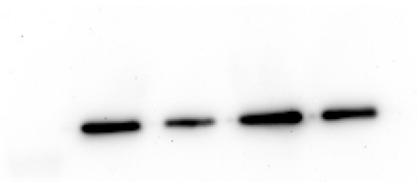

UBE2A (17kDa)

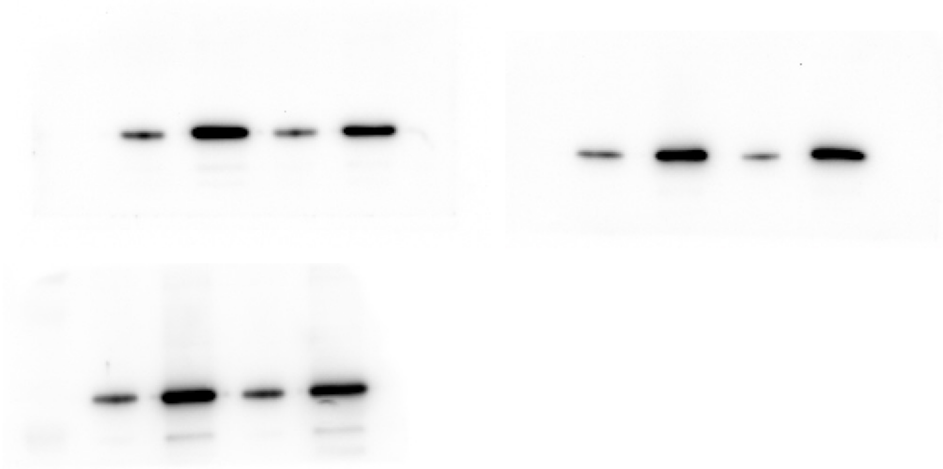

GAPDH (37kDa)

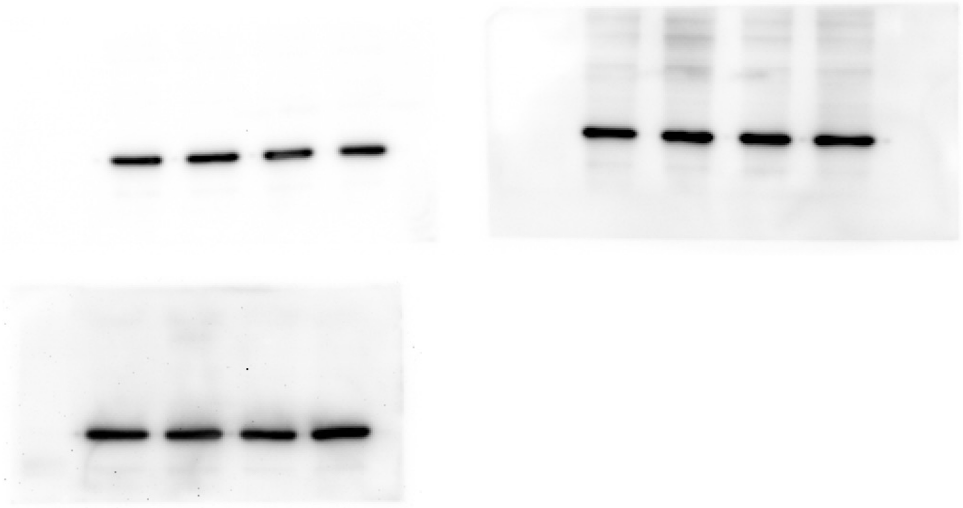

Fig6.I

UBE2A (17kDa)

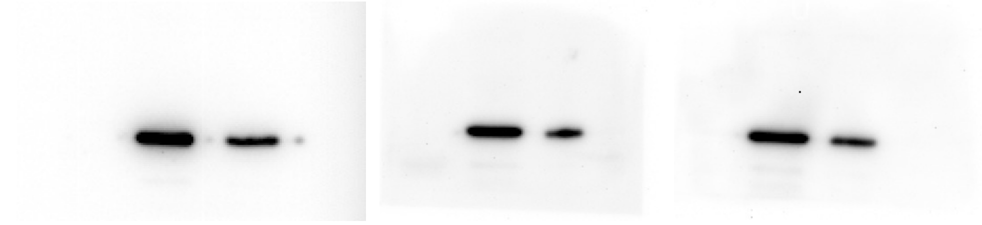

MDM2 (60kDa)

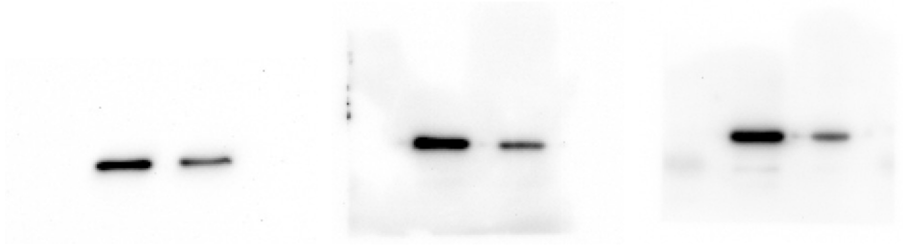

P53 (53kDa)

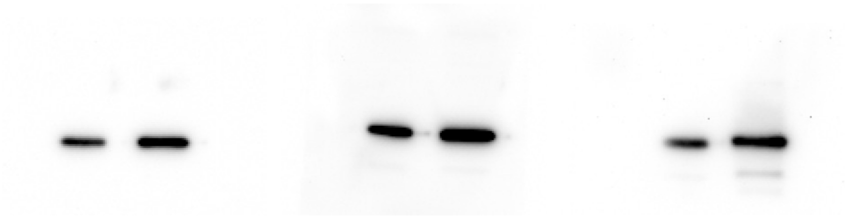

Bcl2 (26kDa)

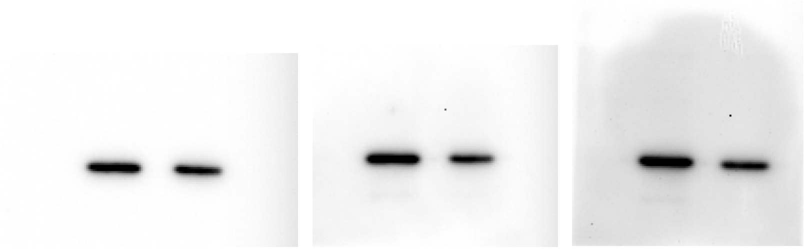

Bax (21kDa)

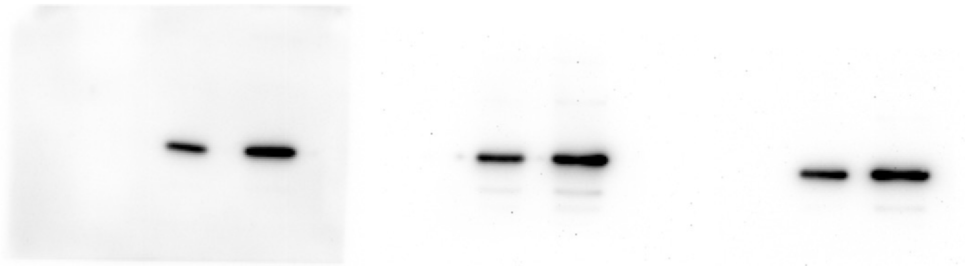

GAPDH (37kDa)

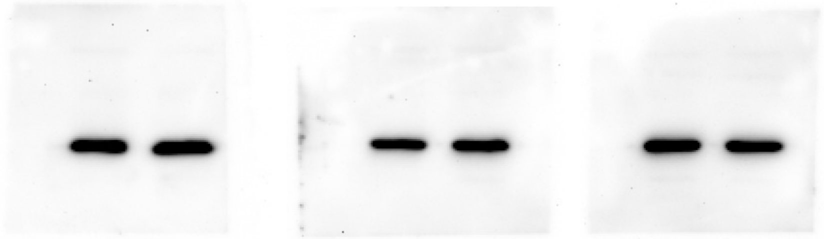

UBE2A (17kDa)

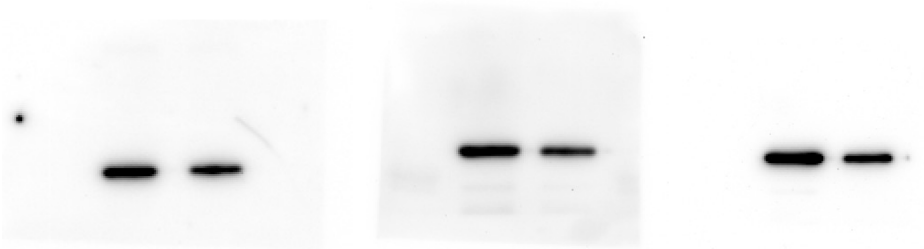

MDM2 (60kDa)

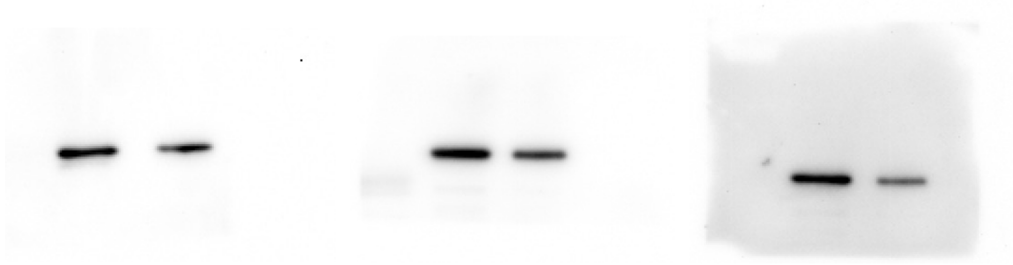

P53 (53kDa)

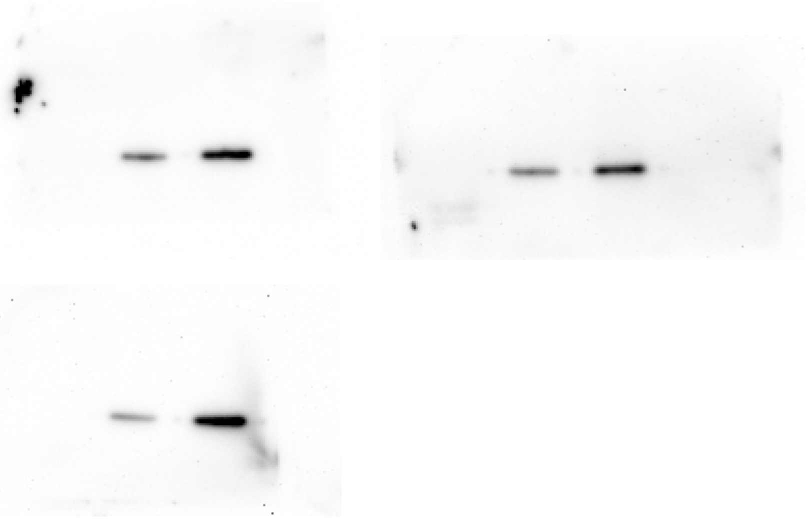

Bcl2 (26kDa)

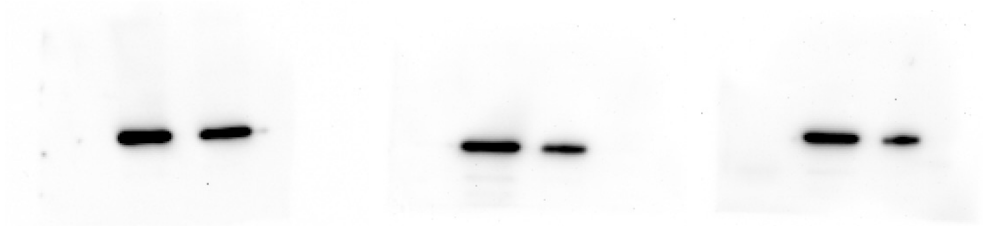

Bax (21kDa)

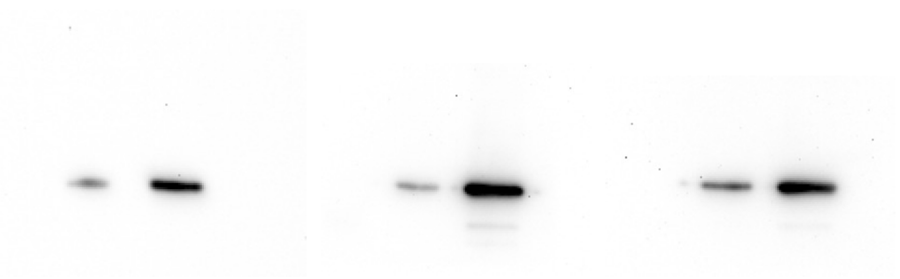

GAPDH (37kDa)

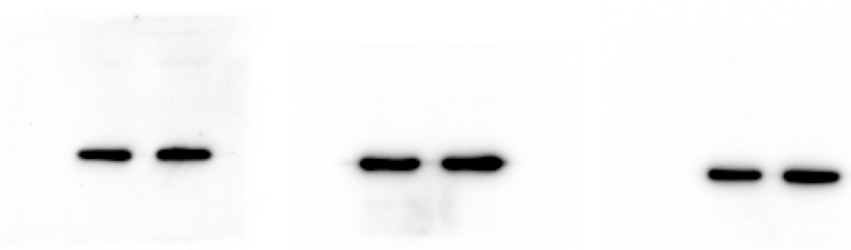

Supplement: Supplementary file 1 — Original Data File [file 41420_2022_866_MOESM1_ESM.pdf]
